# DNA Damage Following Acute Aerobic Exercise: A Systematic Review and Meta-analysis

**DOI:** 10.1007/s40279-019-01181-y

**Published:** 2019-09-16

**Authors:** Despoina V. Tryfidou, Conor McClean, Michalis G. Nikolaidis, Gareth W. Davison

**Affiliations:** 1 Sport and Exercise Sciences Research Institute, Ulster University, Shore Road, Newtownabbey, Northern Ireland UK; 2 Department of Physical Education and Sports Science at Serres, Aristotle University of Thessaloniki, Serres, Greece

## Abstract

**Background:**

Exercise is widely recognised for its health enhancing benefits. Despite this, an overproduction of reactive oxygen and nitrogen species (RONS), outstripping antioxidant defence mechanisms, can lead to a state of (chronic) oxidative stress. DNA is a vulnerable target of RONS attack and, if left unrepaired, DNA damage may cause genetic instability.

**Objective:**

This meta-analysis aimed to systematically investigate and assess the overall effect of studies reporting DNA damage following acute aerobic exercise.

**Methods:**

Web of Science, PubMed, MEDLINE, EMBASE, and Scopus were searched until April 2019. Outcomes included (1) multiple time-points (TPs) of measuring DNA damage post-exercise, (2) two different quantification methods (comet assay and 8-oxo-2′-deoxyguanosine; 8-OHdG), and (3) protocols of high intensity (≥ 75% of maximum rate of oxygen consumption; *V*O_2-max_) and long distance (≥ 42 km).

**Results:**

Literature search identified 4316 non-duplicate records of which 35 studies were included in the meta-analysis. The evidence was strong, showcasing an increase in DNA damage immediately following acute aerobic exercise with a large-effect size at TP 0 (0 h) (SMD = 0.875; 95% CI 0.5, 1.25; *p* < 0.05). When comparing between comet assay and 8-OHdG at TP 0, a significant difference was observed only when using the comet assay. Finally, when isolating protocols of long-distance and high-intensity exercise, increased DNA damage was only observed in the latter. (SMD = 0.48; 95% CI − 0.16, 1.03; *p* = 0.15 and SMD = 1.18; 95% CI 0.71, 1.65; *p* < 0.05 respectively).

**Conclusions:**

A substantial increase in DNA damage occurs immediately following acute aerobic exercise. This increase remains significant between 2 h and 1 day, but not within 5–28 days post-exercise. Such an increase was not observed in protocols of a long-distance. The relationship between exercise and DNA damage may be explained through the hormesis theory, which is somewhat one-dimensional, and thus limited. The hormesis theory describes how exercise modulates any advantageous or harmful effects mediated through RONS, by increasing DNA oxidation between the two end-points of the curve: physical inactivity and overtraining. We propose a more intricate approach to explain this relationship: a multi-dimensional model, to develop a better understanding of the complexity of the relationship between DNA integrity and exercise.

**Electronic supplementary material:**

The online version of this article (10.1007/s40279-019-01181-y) contains supplementary material, which is available to authorized users.

## Key Points

**Table:** 

Acute exercise can damage single-stranded DNA, and DNA repair likely occurs within at least 3 days.
Multiple factors affect the extent of exercise-induced DNA damage and its repair.
An elaborate, multi-dimensional approach should be considered to fully understand the complex relationship between exercise, reactive oxygen and nitrogen species, and DNA damage.

## Introduction

Exercise is widely regarded as a primary conduit to a proficient state of health, and there is now ample evidence from both observational studies and randomised trials to postulate that regular exercise is a contributing factor in the prevention of cardiovascular disease, cancer, diabetes, and other chronic conditions, as well as reducing the risk of all-cause mortality [1, 2].

Despite this paradigm, multiple studies have established a link between strenuous and/or exhaustive exercise, and the increased formation of reactive oxygen and nitrogen species (RONS) [3]. RONS are generated endogenously in most aerobic organisms by an incomplete reduction of oxygen, and mainly via the mitochondrial electron transport chain during cellular respiration [4]. It is currently well understood that between 0.12 and 2% of the oxygen utilised by mitochondria during normal respiration is not converted to water (tetravalent reduction), but instead is reduced to the superoxide anion (O_2_^·−^), which can subsequently be reduced to hydrogen peroxide (H_2_O_2_) and further to the more potent hydroxyl free radical (OH^·^) [5, 6]. However, of note, the percentage estimation of total oxygen consumption in mitochondrial RONS production refers primarily to the in vitro-based experiments performed by Chance and colleagues [7]; as such, the production of O_2_^−^ in vivo may indeed be much less [8–10].

RONS are often implicated in complex molecular mechanisms designed to explain the process of human ageing and associated chronic disease states [11]. Molecules such as lipid, protein, and DNA are known vulnerable targets of RONS attack, and therefore, can be oxidatively modified [12]. Oxidative free radical attack and subsequent damage to DNA in particular, is of prime biomedical importance and interest, as if left unrepaired, significant DNA alterations (e.g., chromosomal rearrangement, base damage, and strand breaks) may lead to rapid ageing, mutagenesis, and ultimately carcinogenesis [13–15]. Paradoxically, although excessive RONS production may be implicated in the pathology of numerous diseases [16], when produced in moderate/low amounts (i.e., not inducing a state of oxidative stress defined as an ‘imbalance between oxidants and antioxidants in favor of the oxidants, leading to a disruption of redox signaling and control and/or molecular damage’) [17], they act as key intracellular signalling molecules regulating a host of physiological and biological processes [11, 18]. RONS are generated in skeletal muscle, and play a key role in skeletal muscle adaptation to aerobic exercise training [19, 20]. In vitro work has shown myotubes exposed to hydrogen peroxide (exogenous), which increases the expression of peroxisome proliferator-activated receptor (PPAR-γ), and peroxisome-gamma co-activator-1 alpha (PGC-1α), whereas further exposure to *N*-acetylcysteine, an antioxidant, impeded its activity [21]. PGC-1α, which is induced by AMP kinase (AMPK), is a signalling pathway involved in adaptation to endurance exercise leading to mitochondrial biogenesis [18]. Similarly, in vivo work has demonstrated that antioxidant supplementation can hinder essential training adaptation mechanisms in humans. A study administrating 1 g of vitamin C per day during 8 weeks of training (3 days/week at 65–80% *V*O_2-max_; 5% increase every 2 weeks) resulted in a decreased expression of PGC-1α and mitochondrial transcription factor A, both of which are key transcription factors involved in mitochondrial biogenesis [22].

Mechanistically, there are several ways free radicals can be generated during exercise. While exercising, the energy requirements in the body greatly increase, leading to a substantially higher rate of oxygen uptake up to 15-fold, and in active muscle, the oxygen flux may increase to approximately 100-fold compared to resting values [23, 24]. The primary radical species produced by the contracting skeletal muscle are O_2_^·−^ and nitric oxide (NO) [25]. When electron transfer occurs normally through the electron mitochondrial transport chain to reduce oxygen to water, approximately 1–3% of all electrons are leaked resulting in the formation of O_2_^·−^ by adding one electron to molecular oxygen [13, 26]. Apart from the mitochondria, there are enzymatic sources that contribute substantially to free radical production such as nicotinamide adenine dinucleotide phosphate (NADPH) oxidase, the enzyme which catalyses the one electron reduction of molecular oxygen (reaction 1) upon the activation of phagocytosis [13, 27].

$${\text{HO}}_{2}^{ \cdot - } \rightleftarrows {\text{O}}_{2}^{ \cdot - } + {\text{H}}^{ + } {\mkern 1mu} {\mkern 1mu} ({\text{reaction}}{\mkern 1mu} {\mkern 1mu} 1).$$

Central to these mechanisms is the generation of superoxide anion (O_2_^·−^; one-electron reduction), and subsequently, produced through the superoxide dismutases (SODs), hydrogen peroxide (H_2_O_2_; two-electron reduction) [13]. Following the production of H_2_O_2_, the hydroxyl radical (OH^·^; three-electron reduction) can be produced in the presence of transition metal catalysts, through the Haber–Weiss Fenton reaction (reaction 2) [13, 26]. O_2_^·−^ also reacts with NO (reaction 3) to produce peroxynitrite (ONOO^-^), a highly reactive molecule, that can damage DNA and nitrate proteins [26]:

$${\text{H}}_{ 2} {\text{O}}_{ 2} + {\text{ Fe}}^{ 2+ } \rightleftarrows {\text{Fe}}^{ 3+ } + {\text{ OH}}^{ - } + {\text{ OH}}^{ \cdot } \,\,({\text{reaction\,2}})$$

$${\text{O}}_{ 2}^{ \cdot - } + {\text{NO}}^{ \cdot } \to {\text{ONOO}}^{ - } \left( {\text{reaction\,3}} \right).$$

As RONS accumulate in the cell, from either metabolic signalling (NADPH) pathways or external sources, they are balanced by scavenging antioxidant systems [28]. Under these balanced conditions, RONS are used as signalling molecules or, under unbalanced conditions, can interact with Fe^2+^ through Fenton chemistry, as mentioned, and cause DNA damage due to hydroxyl radicals (OH^·^), which in turn can be attenuated by DNA repair mechanisms. In the case of over-accumulation of such DNA damage and insufficient repair, it is conceivable to suggest that rapidly dividing cells may promote a mutational profile leading to disease. However, per their signalling role, RONS and DNA damage can trigger physiological programmed cell death (apoptosis) by activating p53 to prevent mutagenesis/carcinogenesis [28]. Therefore, it is important to differentiate whether cell death is caused by oxidative stress (i.e., DNA damage), which can be avoided (scavenging systems and DNA repair mechanisms), or programmed cell death via RONS signalling which could be advantageous when the cell becomes compromised, as a result of DNA damage [28]. RONS are therefore, important molecules involved in the fate of the cell’s destiny as they regulate crucial processes such as growth, differentiation, and cell death [29]. Once DNA is damaged, it is normally repaired by mechanisms such as base excision repair (BER), nucleotide excision repair (NER), or through a process of homologous recombination (HR) or nonhomologous end joining (NHEJ); the type of DNA repair will depend on the mechanism and the extent of the damage [30] (Electronic Supplementary Material Figure S1).

Exercise represents an intriguing model to examine the dynamic role of RONS from both a physiological and pathological perspective. Evidence suggests that only exhaustive (long-distance) and/or strenuous exercise (high-intensity maximal exercise, marathons, triathlons, and overtraining) can induce detrimental DNA alterations, if left unrepaired [18, 31]. However, during low or moderate intensity and distance exercise, RONS may serve to act as signalling molecules responsible for the initiation of exercise and skeletal muscle adaptation [19, 31, 32], as often conceptualised through the hormesis theory.

The aim of this work is to systematically investigate data reporting DNA damage following acute aerobic exercise, and perform a meta-analysis to examine the overall effect from these studies. There are discrepancies regarding exercise intensity and that it necessarily needs to be very exhaustive/strenuous to cause oxidative damage and/or stress, and this review will aim to elucidate this. Furthermore, the possible physiological and/or pathological consequences of exercise-induced DNA damage need to be considered in relation to the exercising individual in line with a new proposed multi-dimensional model. This is the first meta-analysis aimed to investigate the relationship between DNA damage and exercise.

## Methods

### Search Strategy

According to the PRISMA (Preferred Reporting Items for Systematic reviews and Meta-Analyses) guidelines [33], a detailed search was conducted to identify all relevant studies (including a range of publication from 1900 to April 2019) across the following five databases: Web of Science, PubMed, MEDLINE, EMBASE, and Scopus. Searching was limited to articles published in English and the filter “in humans” was applied on PubMed, MEDLINE, and EMBASE.

### Inclusion/Exclusion Criteria

All published studies were checked for the following criteria: (1) the study was a full report published in a peer-reviewed journal; (2) the study assessed humans; and (3) the keyword combination referred to the following terms (used in all possible combinations): exercise, exercis*, exercise training, endurance, exhaustive, exercise-induced, acute exercis*, physical activity, DNA, nucleoid DNA, deoxyribonucleic acid, 8-hydroxy-2′-deoxyguanosine, 8-hydroxy-2-deoxyguanosine, 8-oxo-7,8-dihydro-2′-deoxyguanosine, 8-oxo-7,8-dihydro-2-deoxyguanosine, 8-hydroxy-2-deoxy guanosine, 8-hydroxydeoxyguanosine, 8-oxoguanine, 8-hydroxyguanosine, 8-oxo-2-deoxyguanosine, 8-OHdG, 8OHdG, 8-OH-dG, 8-OHG, 8-oxo-dG, 8-oxodG, 8-oxo-dG, 8-oxo-G, damage, oxidative damage, oxidative stress. Note that for the purposes of this review, we used the term *DNA damage* to encompass DNA single-strand breakage and nucleotide base oxidation.

One investigator initially reviewed records generated from all databases and applied the inclusion/exclusion criteria to identify eligible studies for inclusion; these were then agreed with at least three of the authors. The inclusion/exclusion criteria are shown in Table 1. To note, acute exercise was defined as aerobic exercise performed over a short period of time, but could also extend up to 1–3 days of a marathon event. To minimise the limitation of various biological samples, studies utilising urine, red blood, and muscle cells were also excluded. Please see Electronic Supplementary Material Table S1 for information and details of excluded studies.

**Table 1:** Inclusion/exclusion criteria

Criteria	Include	Exclude
Participants	Humans	Animals
Age group	18–70	Children, elderly
Exercise protocol	Acute, aerobic	Anaerobic, training (chronic)
Sample	White blood cells (leukocytes, lymphocytes, PBMC)	Urine, red blood cells (erythrocytes), muscle
Outcome measure	SBs (%), tail damage (%), tail length, tail moment, 8-OHdG	Double SBs

*SBs* strand breaks, *8*-*OHdG* 8-hydroxy-2’–deoxyguanosine, *PBMC* peripheral blood mononuclear cells

### Data Extraction

A general extraction form was used, once the number of included studies was finalised. Characteristics of the participants (sample size, age, and sex), the exercise protocol (distance and intensity), assayed biomarkers, and methods of DNA quantification used were extracted by one investigator. The outcome measure, DNA damage, was expressed using multiple descriptors, and with regard to the comet assay, these were: DNA in the tail (%); DNA migration (μm) (otherwise known as tail length); tail moment (also known as olive tail moment) which is the product of tail (%) and tail length [34]. The biomarker used was 8-OHdG. Due to variations in the analytical approach, high-performance liquid chromatography (HPLC) or enzyme-linked immunosorbent assay (ELISA), 8-OHdG (pg/ml), and 8-OHdG/10^5^ dG are also reported. The tail DNA (%), DNA migration (μm) or tail length and tail moment correspond to the comet assay and 8-OHdG (ng/ml) or (pg/ml) and 8-OHdG/10^5^ dG to HPLC or ELISA methods. In reference to the comet assay, where multiple image descriptors were reported by one study, the authors used tail (%), as this is regarded as the most sensitive descriptor/parameter compared to tail moment or length [35]. Data were collected as means and standard deviation (SD) or standard error of the mean (SEM). Graph digitizer software (DigitizeIt, Braunschweig, Germany) and WebPlotDigitizer (Web Plot Digitizer, V.4.2. Texas, USA: Ankit Rohatgi, 2019) were used to obtain data from studies where data were only presented in a figure format. In two studies [36, 37], data were not extractable and, therefore, not included in the meta-analysis.

Numerous studies included heterogeneous groups of participants: trained or untrained, young or old, sport-specific volunteers (such as swimmers, rowers, and runners), physically active and sedentary participants, and a few studies compared men and women. Furthermore, three studies [38–40] used more than one parameter to quantify DNA damage. Finally, some studies measured DNA damage at only one time-point (TP), while other studies included multiple post-exercise measures of DNA damage following exercise. Table 2 details corresponding TPs for each investigation.

**Table 2:** Individual time-points (TP) of measures of DNA damage after exercise for each investigation

Study	TP 0 (0 h)	TP 1 (15 min–1 h)	TP 2 (2 h)	TP 3 (3 h)	TP 4 (4–6 h)	TP 5 (1 day)	TP 6 (2 days)	TP 7 (3 days)	TP 8 (4 days)	TP 9 (5 days)	TP 10 (6–7 days)	TP 11 (14–28 days)
Bloomer et al. [59]	0 h											
Briviba et al. [49]		20 min										
Davison et al. [65]	0 h	1 h										
Fogarty et al. [67]	0 h											
Fogarty et al. [66]	0 h											
Harms-Ringdahl et al. [50]		1 h										
Hartmann et al. [70]	0 h				6 h	1d	2d	3d	4d			
Hartmann et al. [68]						1d						
Hartmann et al. [69]	0 h					1d	2d	3d	4d	5d		
Inoue et al. [56]	0 h											
Itoh et al. [60]	0 h	1 h				1d						
Kim et al. [79]	0 h							3d				
Liu et al. [38]	0 h											
Mastaloudis et al. [51]	0 h					1d	2d	3d	4d	5d	6d	
Meihua et al. [58]	0 h											
Møller et al. [34]	0 h					1d	2d					
Niess et al. [39]		15 min				1d						
Niess et al. [71]		1 h										
Paik et al. [72]	0 h	1 h										
Peters et al. [73]	0 h			3 h								
Pittaluga et al. [53]		30 min				1d						
Revan [61]	0 h	30 min										
Ryu et al. [40]	0 h							3d				
Sacheck et al. [62]						1d						
Sardas et al. [55]						1d						
Saritaş et al. [63]	0 h					1d						
Sato et al. [64]	0 h	1 h				1d	2d					
Shi et al. [57]	0 h			3 h		1d						
Tanimura et al. [74]	0 h	1 h	2 h	3 h	4 h							
Tanimura et al. [54]						1d						
Tsai et al. [75]	0 h					1d		3d			7d	14d
Turner et al. [76]	0 h					1d					7d	28d
Wagner et al. [77]		20 min				1d				5d		19d
Williamson et al. [80]	0 h											
Zhang et al. [78]			2 h		6 h	1d						

### Data Analysis

The primary outcome was defined as DNA oxidative damage before and following exercise at TP 0 (0 h) grouped by method of DNA damage quantification (1) comet assay and (2) 8-OHdG. Secondary outcomes included: (3) high intensity (≥ 75% of maximum rate of oxygen consumption; *V*O_2-max_) and (4) long distance (≥ 42 km) as different exercise protocols were measured, and finally (5), DNA damage at further time-points 1–11 (ranging from 15 min to 28 days).

### Quality Assessment

To assess the quality of included studies, the risk of bias was assessed by one investigator using the 12 criteria (rating: yes, no, and unsure) recommended by the Cochrane Back Review Group (Table 3) [41]. The criteria assess risk of bias using the five following categories: selection bias; performance bias; attrition bias; reporting bias, and detection bias. However, due to the inherent difficulties in blinding participants to exercise treatments, 7 of the 12 criteria were not applicable, and as such not included. These were: adequate method of randomization; allocation concealment; outcome assessor blinding; participant and provider blinding; similarity or not of co-interventions; and intention-to-treat analysis. In contrast, two additional sources of bias, smoking and training status, were included as criteria given their potential to influence exercise responses. Following these modifications, the maximum score that studies could gather was seven, with the lowest scores indicating high risk of bias and higher scores indicating lower risk of bias. To establish a clearer overall assessment of bias, a high-, moderate-, and low-risk scale was developed according to how studies scored. Therefore, the following ranges were developed: 1–3 = high risk, 4–5 = moderate, and 6–7 = low risk.

**Table 3:** Quality assessment for risk of bias of the included studies using the criteria recommended by the Cochrane back review group

Study	Selection bias	Performance bias	Attrition bias	Reporting bias		Study-specific		Total score (maximum 7)
	Similar baseline characteristics	Acceptable compliance	Acceptable and described drop-out rate	No selective outcome reporting	Similar timing of outcome assessment	Training status reported	Smoking status reported	
Asami et al. [36]^a^	Unsure	Yes	No	Yes	Yes	No	Yes	4
Bloomer et al. [59]	Yes	Yes	Yes	Yes	Yes	Yes	Yes	7
Briviba et al. [49]	No	Yes	Yes	Yes	Yes	No	No	4
Davison et al. [65]	Yes	Yes	Yes	Yes	Yes	No	Yes	6
Fogarty et al. [67]	Yes	Yes	Yes	Yes	Yes	Yes	Yes	7
Fogarty et al. [66]	Yes	Yes	Yes	Yes	Yes	No	No	5
Harms-Ringdahl et al. [50]	No	Yes	Yes	Yes	Yes	No	Yes	5
Hartmann et al. [70]	No	Yes	Yes	Yes	Yes	Yes	Yes	6
Hartmann et al. [68]	Yes	Yes	Yes	Yes	Yes	No	Yes	6
Hartmann et al. [69]	No	Yes	Yes	Yes	Yes	Yes	Yes	6
Inoue et al. [56]	No	Yes	Yes	Yes	Yes	Yes	Yes	6
Itoh et al. [60]	Yes	Yes	Yes	Yes	Yes	Yes	Yes	7
Kim et al. [79]	Yes	Yes	Yes	Yes	Yes	Yes	Yes	7
Liu et al. [38]	Yes	Yes	Yes	Yes	Yes	No	Yes	6
Mastaloudis et al. [51]	No	Yes	Yes	Yes	Yes	No	Yes	5
Meihua et al. [58]	Yes	Yes	Yes	Yes	Yes	No	Yes	6
Møller et al. [34]	No	Yes	Yes	Yes	Yes	No	No	4
Niess et al. [39]	Yes	Yes	Yes	Yes	Yes	Yes	No	6
Niess et al. [71]	Yes	Yes	Yes	Yes	Yes	Yes	No	6
Orlando et al. [48]^a^	Yes	Yes	Yes	No	Yes	Yes	No	5
Paik et al. [72]	Yes	Yes	Yes	Yes	Yes	No	No	5
Peters et al. [73]	Yes	No	No	Yes	Yes	Yes	No	4
Pittaluga et al. [53]	Yes	Yes	Yes	Yes	Yes	Yes	Yes	7
Revan [61]	Yes	Yes	Yes	Yes	Yes	Yes	Yes	7
Roh et al. [37]^a^	Yes	Yes	Yes	Yes	Yes	Yes	Yes	7
Ryu et al. [40]	Yes	Yes	Yes	Yes	Yes	Yes	Yes	7
Sacheck et al. [62]	No	Yes	Yes	Yes	Yes	Yes	Yes	6
Sardas et al. [55]	Yes	Yes	Yes	Yes	Yes	Yes	Yes	7
Saritaş et al. [63]	Yes	Yes	Yes	Yes	Yes	No	Yes	6
Sato et al. [64]	Yes	Yes	Yes	Yes	Yes	Yes	Yes	7
Shi et al. [57]	Yes	Yes	Yes	Yes	Yes	Yes	Yes	7
Tanimura et al. [74]	Yes	Yes	Yes	Yes	Yes	No	Yes	6
Tanimura et al. [54]	No	Yes	Yes	Yes	Yes	Yes	Yes	6
Tsai et al. [75]	Yes	Yes	Yes	Yes	Yes	No	No	5
Turner et al. [76]	Yes	No	No	Yes	Yes	No	Yes	4
Wagner et al. [77]	Yes	Yes	Yes	Yes	Yes	Yes	No	6
Williamson et al. [80]	Yes	Yes	Yes	Yes	Yes	Yes	Yes	7
Zhang et al. [78]	Yes	Yes	Yes	Yes	Yes	Yes	Yes	7

^a^Not included in meta-analyses

### Statistical Analysis

*Assessment of effect size* Meta-analyses were calculated using Comprehensive Meta-Analysis (Version 3.3.070, NJ:USA: Biostat, Inc). A random-effects model was used, since it assumes statistical heterogeneity among studies and that studies represent a random sample of effect sizes that could have been observed [42, 43]. Standardised mean differences (SMD) adjusted with Hedges’ g and 95% confidence intervals (CI) were calculated as the difference in means before and after exercise divided by the pooled standard deviation [43]. Where studies did not report standard deviations, these were calculated from standard errors [42]. The SMD measure was used to express effect size, the magnitude of which was calculated using Cohen’s categories: (1) small: SMD = 0.2–0.5, (2) medium: SMD = 0.5–0.8, and (3) large: SMD > 0.8 [44, 45]. A positive SMD measure was considered to show increased DNA damage after exercise compared to rest, whereas a negative SMD measure would show greater DNA damage at rest in comparison to after exercise. The overall effect was assessed using *Z* scores with a set significance level of *p* < 0.05.

*Assessment of heterogeneity* The Chi-square Cochran’s *Q* test and the *I*^2^ statistic were used for the assessment of statistical heterogeneity among studies. The Chi-square test assesses whether the observed differences in results are compatible with chance alone and a *p* value ≤ 0.10 was considered to display significant heterogeneity [42]. Furthermore, the *I*^2^ statistic was used to quantify inconsistency across studies, with (1) *I*^2^ = 0–30% showing no heterogeneity, (2) *I*^2^ = 30–49% showing moderate heterogeneity, (3) *I*^2^ = 50–74% showing substantial heterogeneity, and (4) *I*^2^ = 5–100% showing considerable heterogeneity [42].

*Subgroup and sensitivity analysis* Subgroup analyses were performed for multiple time-points of DNA damage quantification after exercise grouped by different methodologies in DNA quantification (comet assay versus 8-OHdG), and according to the exercise protocol: high intensity (≥ 75% *V*O_2-max_) versus long distance (≥ 42 km). To assess the robustness of the significant outcome data, sensitivity analysis was planned by excluding studies with high risk of bias.

*Publication bias* Publication bias was assessed, when at least ten studies were included in the meta-analyses, by visually analysing funnel plots. In general, asymmetrical funnel plots were considered to indicate high risk of publication bias, while symmetrical funnels plots were considered to indicate low risk [46].

## Results

### Literature Search

**Fig. 1:**
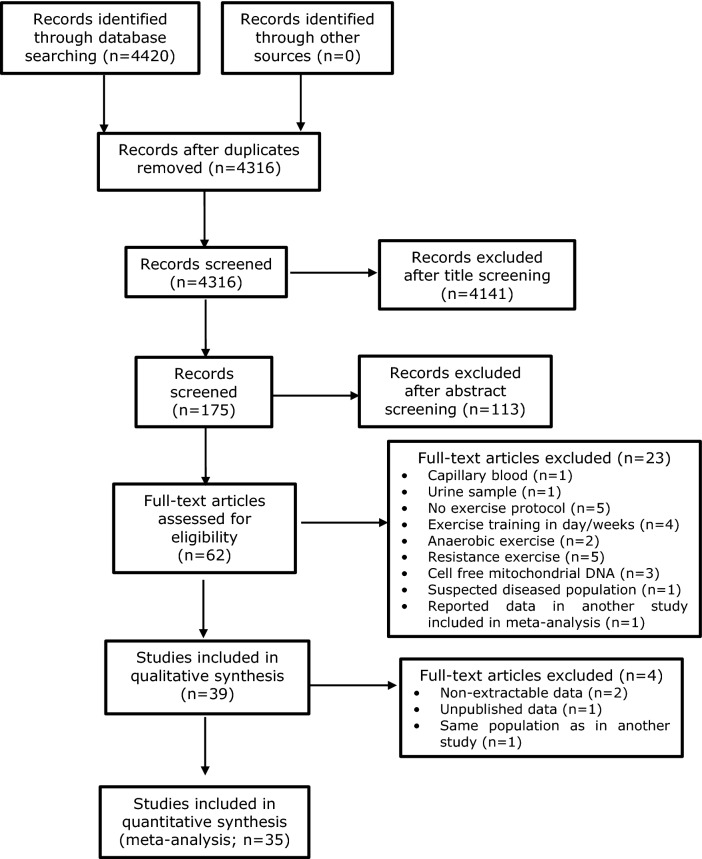
PRISMA flow diagram displaying the electronic search and selection process

The number of articles identified from all electronic database searches and the selection process is shown in Fig. 1. Four thousand four hundred and twenty records (4420) were retrieved in the database search, one hundred and four (104) of which were duplicates. Four thousand one hundred and forty-one articles (4141) were excluded after title screening, leaving one hundred and seventy-five (175) records for abstract screening. One hundred and thirteen (113) records were excluded after abstract screening and sixty-two (62) full-text articles were assessed for eligibility. Twenty-three (23) full-text articles were excluded due to various reasons (detailed in Electronic Supplementary Material Table S1). The most common reason for study exclusion was the exercise protocol not consisting of acute and aerobic exercise. Thirty-nine studies (39) were included in the qualitative analysis, out of which one (1) was excluded due to same sample size [47], one had unpublished data [48], and two (2) due to non-extractable data [36, 37], and therefore, thirty five (35) were included in the quantitative analysis.

### Study Characteristics

The characteristics of each study including participants, exercise protocol, sample source, biomarker, quantification technique, and results are presented in Table 4.

**Table 4:** Characteristics of the included studies and relevant outcomes

Study	Participants	Exercise protocol	Sample	Biomarker	Technique	Findings
Møller et al. [34]	12 Healthy subjects (7 males and 5 females); MA ± SD = 26.1 ± 4.9 y/o	Maximal bicycle exercise (*V*O_2-max_ test) under normal conditions and at 4559 m (high-altitude hypoxia) for 3 days	Lymphocytes	SBs,FPG-s s, ENDO III-s s	Comet assay	Increased SBs at hypoxia on all 3 days; ENDO III-s s increased on day 3 at hypoxia; FPG-s s remained unchanged
Fogarty et al. [66]	10 Healthy males; MA ± SD = 23 ± 4 y/o	Treadmill test to exhaustion	Lymphocytes	Tail DNA (%)	Comet assay	DNA damage increased 63% (*p* < 0.05) after exercise compared to rest
Sardas et al. [55]	12 Male rowers (MA ± SD = 22 ± 3.8 y/o) and 11 PE students (MA ± SD = 21.8 ± 3.8 y/o)	Three-staged exercise test on a rowing (rowers) and bicycle ergometer (PE students)	Lymphocytes	Tail DNA (%)	Comet assay	DNA damage was increased 24 h post exercise compared to pre-exercise in both rowers and PE students but overall pre- and post-exercise damage was significantly higher in rowers compared to PE students
Reichhold et al. [47]^a^	28 Male triathletes; MA ± SD = 32.7 ± 6.3 y/o	Ironman triathlon (3.8 km swim, 180 km cycle, 42 km run)	Lymphocytes	Tail DNA (%)	Comet assay	The % of SBs decreased significantly immediately post-race, then increased 1 d post-race and declined again 5 d after the race Between days 5 and 19 post-race the levels of strand breaks decreased further below initial levels
Hartmann et al. [70]	2 Males (20 and 30 y/o) and 1 female (32 y/o)	Treadmill test to exhaustion	White blood cells	DNA migration (μm)	Comet assay	No increase in DNA migration was seen immediately after the test but a significant increase in DNA migration was found after 6, 24 and 48 h.
Liu et al. [38]	8 Males athletes; MA ± SD = 21.35 ± 1.04	Treadmill exercise to exhaustion	Peripheral blood cells	Tail length; Olive tail moment	Comet assay	Compared to before exercise, both tail length and olive tail moment significantly rose after exercise (*p* < 0.001)
Inoue et al. [56]	9 Swimmers (MA ± SD = 20.1 ± 1.2 y/o) and 9 distance runners (MA ± SD = 20.9 ± 2.1 y/o)	1500 m for 90 min (swimmers) and 15 km distance running for 70 min (runners)	Lymphocytes	8-OHdG	HPLC	8-OHdG decreased significantly after swimming, but no significant change was observed after running
Sacheck et al. [62]	8 Young (MA ± SD = 25.4 ± 2.2 y/o) and 8 older (MA ± SD = 69.3 ± 3.5 y/o)	Downhill running on a treadmill for 45 min at 75% *V*O_2-max_	Leukocytes	8-OHdG	HPLC	No change in 8-OHdG 24 h post-exercise
Revan [61]	14 Healthy males; MA ± SEM = 22.3 ± 0.5 y/o	Incremental exercise test to exhaustion on a cycle ergometer	Plasma	8-OHdG	Cayman ELISA Kit	No significant difference in 8-OHdG levels before and after exercise
Tanimura et al. [54]	8 Young untrained (MA ± SD = 23.8 ± 3.2 y/o) and 8 endurance-trained (MA ± SD = 21.1 ± 3.7 y/o) men	Cycling at 75% *V*O_2-max_ 1 h daily for three consecutive days	Lymphocytes	Tail DNA (%)	Comet assay	DNA damage at day 4 was significantly greater than that at day 1 in both groups
Saritaş et al. [63]	22 Healthy trained men; MA ± SEM = 21.45 ± 0.43 y/o	12 min run test	Serum	8-OHdG	ELISA Kit	8–OHdG levels were not different between the time periods (before exercise, immediately after exercise and 24 h after the exercise) (*p* > 0.05)
Mastaloudis et al. [51]	5 Males (MA ± SEM = 39 ± 8 y/o) and 5 females (MA ± SEM = 35 ± 4 y/o)	50 km ultramarathon	Leukocytes	Tail DNA (%)	Comet assay	On average, in both placebo and experimental groups, DNA damage increased at mid-race but returned to baseline values by the end of the race
Harms-Ringdahl et al. [50]	15 Healthy untrained males and females (7 females and 8 males; MA ± SD = 30.5 ± 6.9 y/o	20 min cycling at 80% of maximum heart rate	Serum	8-OHdG	ELISA Kit	Levels of 8-OHdG increased 42% after the exercise
Hartmann et al. [69]	3 Male and 3 female athletes (aged between 21-33 y/o)	Short-distance triathlon competition (1.5 km swimming, 40 km cycling, 10 km running)	Lymphocytes	Tail moment (DNA migration)	Comet assay	Increase in DNA migration was seen at 24 h post-exercise, whereas at 48 h the values were lower compared to 24 h but higher than the pre-exercise values. At 72 h, the maximum increase in DNA migration was found and baseline values were still elevated after 120 h
Fogarty et al. [67]	12 Healthy males; MA ± SD = 23 ± 4 y/o)	Bike test at 40,70 and 100% of *V*O_2-max_	Leukocytes	Tail length	Comet assay	Increase in DNA damage observed at moderate (70% VO_2_max) and high-intensity exercise (100% VO_2_max) compared to rest
Tanimura et al. [74]	15 Healthy sedentary males; MA ± SD = 23.7 ± 1.1 y/o	Cycling at 75% *V*O_2-max_ for 1 h	Lymphocytes	Tail DNA (%)	Comet assay	There was increased DNA damage at 3 h post-exercise compared to pre-exercise (*p* < 0.05)
Paik et al. [72]	10 Healthy males; MA ± SD = 25.6 ± 0.8 y/o	Treadmill run to exhaustion at 80% *V*O_2-max_	Lymphocytes	Tail DNA (%), tail length, tail moment	Comet assay	There was increased DNA damage measured just prior to the termination of exercise compared to pre-exercise (*p* < 0.05)
Ryu et al. [40]	30 Male runners, 10 in each group; 10 km group (MA ± SD = 36.5 ± 10.9 y/o), 21 km group (MA ± SD = 45.0 ± 7.8 y/o), 45 km group (MA ± SD = 37.9 ± 13.6 y/o)	3 different marathon distances: 10 km,21 km and 45 km	Lymphocytes	Tail DNA (%), tail length, tail moment	Comet assay	Compared to rest and recovery, tail moment was significantly higher in all groups. Also, at 45 km tail moment was found to be higher a post-exercise compared to the 10 km and 21 km (*p* < 0.05). No differences in DNA in tail (%) or tail length was observed
Itoh et al. [60]	8 Untrained males; MA ± SD = 21.8 ± 2.1 y/o	10 km run	Plasma	8-OHdG	ELISA kit	Decreased plasma 8-OHdG levels both immediately and 1 h after the 10-km run compared to resting values (*p* < 0.05)
Tsai et al. [75]	14 Male runners (median age 21, range 20–24 y/o)	42 km marathon race	Peripheral blood mononuclear cells	Tail DNA (%), FPG-s s, ENDO III-s s	Comet assay	SBs increased on day 1; increased levels of FPG-s s were observed immediately and 24 h (day 1) after the race; ENDO III-s s levels also increased immediately post-exercise and on day 7
Bloomer et al. [59]	11 & 6 Aerobically trained men and women; MA ± SD = 23.3 ± 5.2 y/o	Treadmill run for 30 min at 80% *V*O_2-max_	Plasma	8-OHdG	ELISA Kit	No significant difference was observed in 8-OHdG levels before and after exercise
Turner et al. [76]	9 Healthy men; MA ± SD = 46.1 ± 5.3 y/o	233 km ultraendurance race	Peripheral blood mononuclear cells	Tail DNA (%), FPG	Comet assay	Increased SBs immediately and 24 h after the race, compared to baseline (*p* < 0.01); an increase in FPG-dependent oxidative DNA damage was also observed immediately after the race (*p* < 0.05)
Peters et al. [73]	8 Male athletes; MA ± SD = 34.2 ± 2.44 y/o	2.5-h treadmill run at 75% *V*O_2-max_	Lymphocytes	Tail length/DNA migration (μm)	Comet assay	There was no significant increase in DNA strand breaks before and after the exercise (*p* > 0.05)
Zhang et al. [78]	11 Healthy male students aged between 18–20 y/o	Bicycle exercise to exhaustion	White blood cells	Tail length/DNA migration (mm)	Comet assay	Significantly increased DNA migration at 6 h and 24 h compared to pre-exercise (*p* < 0.01)
Wagner et al. [77]	42 Male athletes; MA ± SD = 35.3 ± 7 y/o	Ironman triathlon (3.8-km swimming, 180 km cycling and 42.2-km running)	Lymphocytes	SBs, Tail DNA (%), ENDO III-s s, FPG-s s	Comet assay	SBs decreased post-race; DNA migration increased 1 day post-race due to SBs (*p* < 0.01), then 5 days post-race returned to pre-race levels and decreased further to below the initial levels 19 days post-race (*p* < 0.01)
Sato et al. [64]	15 Male subjects aged 19–29 y/o (7 physically active and 8 sedentary)	50% *V*O_2-max_ of cycle ergometer exercise for 30 min	Leukocytes	8-OHdG	HPLC	No change in physically active subjects but decreased in sedentary subjects
Hartmann et al. [68]	8 Healthy men (29-34 y/o)	Treadmill run to exhaustion	White blood cells	Tail moment	Comet assay	Increase in DNA damage was seen 24 h after the run (mean increase = 35.3 ± 8.3%)
Shi et al. [57]	5 Healthy males (aged 22–38 y/o)	Exercise on a cycle ergometer at 50% *V*O_2-max_ for 10.5 ± 1.3 min	Leukocytes	8-OHdG	HPLC	After aerobic exercise, no significant change in leukocyte 8OHdG level was seen. (However, a significant increase was detected in samples taken 24 h after anaerobic exercise)
Davison et al. [65]	7 Healthy males; MA ± SD = 22.3 ± 4.1 y/o	Treadmill test to exhaustion	Peripheral blood mononuclear cells	Tail moment	Comet assay	An increase in DNA damage was observed after exercise
Asami et al. [36]^a^	23 Healthy males aged 19-50 y/o (10 untrained and 13 trained)	Maximal cycling exercise	Leukocytes	8-OHdG	HPLC	A significant decrease in 8-OHdG levels was observed in the untrained subjects only (*p* < 0.05)
Briviba et al. [49]	10 Subjects for half-marathon (5 males and 5 females; MA ± SD = 43 ± 9 y/o) and 12 subjects for marathon (2 males and 10 females; MA ± SD = 45 ± 10 y/o)	Half-marathon (21.1 km) and a marathon (42.195 km)	Lymphocytes	Tail DNA (%), FPG-s s and ENDO III-s s	Comet assay	No significant changes in the levels of DNA strand breaks in lymphocytes after either race. However, a significant difference was found in the % of ENDO III s in the tail after both races (*p* < 0.05), whereas the % of FPG-s s was slightly increased but not significantly (*p* > 0.05)
Pittaluga et al. [53]	7 Females; MA ± SD = 68.1 ± 2.7 y/o	Exhaustive bout on a cycle ergometer	Serum	8-OHdG	HPLC	No significant difference in 8-OHdG levels before and after exercise
Niess et al. [39]	5 UT and 6 TR	Treadmill run test to exhaustion	Leukocytes	Tail moment/DNA migration(μm)	Comet assay	An increase in DNA migration from 2.31 ± 0.20 (TR) and 2.22 ± 0.16 (UT) at rest to 2.65 ± 0.30 (TR) and 3.00 ± 0.41 tail moment (UT) was observed 24 h after exercise
Niess et al. [71]	12 Male runners; MA ± SD = 27.3 ± 4.1 y/o	Half-marathon (21.1 km)	Leukocytes	DNA migration (μm)	Comet assay	DNA migration rose significantly 24 h after the race, compared to rest (*p* < 0.01)
Williamson et al. [80]	10 Recreationally males; MA ± SD = 22 ± 2 y/o	Treadmill test to exhaustion	Peripheral blood mononuclear cells	Tail DNA (%)	Comet assay	Tail intensity was increased by 18.2% post-exercise (*p* < 0.05)
Orlando et al. [48]^a^	21 Rugby male athletes; MA ± SD = 26 ± 5 y/o	40 min run at 85% of maximum heart rate	Peripheral blood mononuclear cells	Tail DNA (%)	Comet assay	No significances of DNA damage were observed after the exercise bout
Meihua et al. [58]	10 Male athletes; MA ± SD = 21.1 ± 1.13 y/o	*V*O_2-max_ (Bruce protocol)	Peripheral blood cells and plasma	DNA damage index and 8-OHdG	Comet assay and HPLC	Exercise increased DNA damage index as measured by comet assay; plasma 8-OHdG levels also increased following exhaustive exercise (*p* < 0.01)
Roh et al. [37]^a^	10 Male college athletes; MA ± SD = 18.8 ± 0.8 y/o	1 h run at 75% of heart rate reserve	Lymphocytes	Tail DNA (%)	Comet assay	DNA tail (%) increased following exercise (*p* < 0.05)
Kim et al. [79]	11 Amateur male triathletes; MA ± SD = 37.9 ± 6.2 y/o	2 triathlon races (O_2_ and Olympic courses)	Lymphocytes	Tail DNA (%)	Comet assay	In the Olympic course, DNA tail intensity (%) increased after match and decreased after 3 and 6 days of recovery; In the O2 course, tail (%) decreased after match, increased after 3 days, and decreased after 6 days of recovery (*p* < 0.01)

*SBs* strand breaks, *FPG*-*s* *s* formamidopyrimidine glycosylase-sensitive sites, *ENDO III*-*s* *s* endonuclease III-sensitive sites, *8*-*OHdG* 8-hydroxy-2′–deoxyguanosine, *MA* mean age, *SD* standard deviation, *HPLC* high-performance liquid chromatography, *ELISA* enzyme-linked immunosorbent assay, *PE* physical education, *VO*_*2*-*max*_ maximum rate of oxygen consumption; untrained, *UT* trained, TR, *y/o* years old

^a^not included in meta-analyses

*Participants* Participant age ranged from 18 to 70 years old. Five studies included both male and female participants [49–53]. Three studies included groups of untrained and trained subjects [36, 39, 54]; one study [55] used rowers and physical education students, while another [56] used swimmers and runners. Finally, two studies used volunteers participating in multiple running distances [40, 49].

*Biomarkers/Analytical Techniques* With regard to the biomarker and the techniques used to quantify DNA damage, 12 studies used 8-OHdG [36, 50, 53, 56–64] with either HPLC or ELISA. A total of 27 studies used tail DNA (%) or strand breaks, tail length, and tail moment with the comet assay technique [37–40, 48, 49, 51, 52, 54, 55, 65–80].

*Exercise Protocol* There was variation in the chosen exercise protocols, most often involving treadmill exercise and cycling whilst employing different exercise intensities (ranged from 40 to 100% *V*O_2-max_). Eight studies included marathons, half-marathons, or ultra-marathons [40, 49, 51, 69, 71, 75, 76, 79], and three studies [47, 77, 79] involved a triathlon as part of the exercise protocol.

*Quality assessment in individual studies* No study scored in the high-risk bias range (1–3), 11 studies scored in the moderate-risk range (4–5), and the remaining 27 studies scored in the low-risk range (6–7).

### Analysis of Overall Effects

In summary, as seen in Table 5, a significant increase in DNA damage following exercise was observed at time-points 0 (0 h), 2 (2 h), 3 (3 h), 4 (4–6 h), 5 (1 days), and 7 (3 days). No significant differences were found at time-points 1 (15 min–1 h), 6 (2 days), 8 (4 days), 9 (5 days), 10 (6–7 days), and 11 (14–28 days).

**Table 5:** Summary of results from all meta-analyses

Time-point	*N* (sample size)			Effect (Hedges’ g)			*p* value		
	8-OHdG	Comet	Overall	8-OHdG	Comet	Overall	8-OHdG	Comet	Overall
0 (0 h)	109	203	312	0.150	1.140	0.875	0.684	0.000*	0.000*
1 (15 min–1 h)	59	104	163	− 0.523	0.448	0.166	0.226	0.105	0.476
2 (2 h)	–	25	–	–	0.556	–	–	0.047*	–
3 (3 h)	5	19	24	1.154	0.968	1.008	0.066	0.003*	0.001*
4 (4–6 h)	–	28	–	–	1.480	–	–	0.010*	–
5 (1 day)	73	148	221	0.141	2.468	1.113	0.720	0.000*	0.000*
6 (2 days)	15	28	43	− 1.208	0.338	0.231	0.509	0.497	0.630
7 (3 days)	–	85	–	–	0.627	–	–	0.022*	–
8 (4 days)	–	19	–	–	0.691	–	–	0.380	–
9 (5 days)	–	44	–	–	0.315	–	–	0.548	–
10 (6–7 days)	–	33	–	–	0.359	–	–	0.491	–
11 (14–28 days)	–	51	–	–	0.105	–	–	0.801	–
High intensity^a^	33	69	102	0.718	1.571	1.179	0.044*	0.000*	0.000*
Long distance^b^	–	89	–	–	0.437	–	–	0.151	–

^a^≥ 75% *V*O_2-max_ at time-point 0 (0 h) and 5 (1 day)

^b^≥ 42 km at time-point 0 (0 h) and 1 (15 min–1 h)

– not applicable

*significance at *p* < 0.05

#### Overall Effect of DNA Damage After Exercise at TP 0

**Fig. 2:**
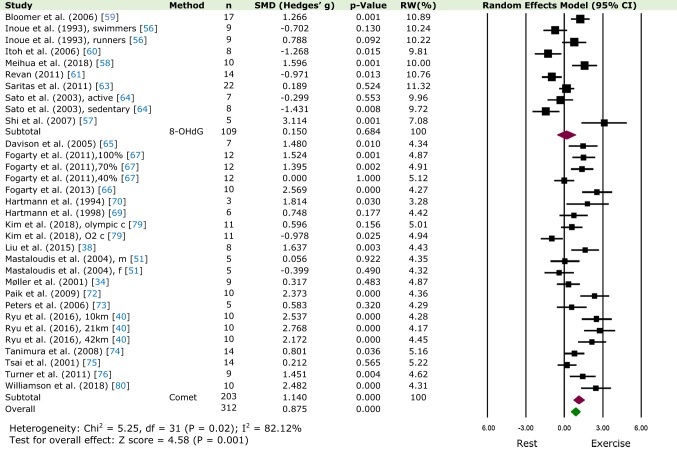
Relative weight (RW) standardised mean difference (SMD) and 95% CI (Hedges’ g adjusted) of DNA damage compared between rest and after an exercise bout at time-point 0 (0 h). Values for individual trials and pooled data (random model) are shown and grouped by method of quantification. *c* course, *m* males, *f* females

For DNA damage after exercise at TP 0 (0 h), data were available from 24 studies, with a total number of 312 participants. As seen in Fig. 2, compared to rest, there was a significant increase in DNA damage after exercise (SMD = 0.875; 95% CI 0.5, 1.25; *p* < 0.05). Heterogeneity among studies was found to be considerable (*χ*^2^ = 5.25, *p* = 0.02, *I*^2^ = 82.12%).

#### Comet Assay vs 8-OHdG at TP 0

Similarly, as shown in Fig. 2, for studies utilizing only the comet assay with 203 participants, DNA damage remained significantly higher after exercise at TP 0 (0 h) compared to rest (SMD = 1.14; 95% CI 0.7, 1.58; *p* < 0.05). Moreover, although the number of studies using the 8-OHdG biomarker was considerably less, with 109 participants, no change in DNA damage compared to rest using this assay was observed (please see Fig. 2; SMD = 0.15; 95% CI − 0.58, 0.88; *p* = 0.68).

#### High-Intensity Exercise (≥ 75% *V*O_2-max_)

**Fig. 3:**
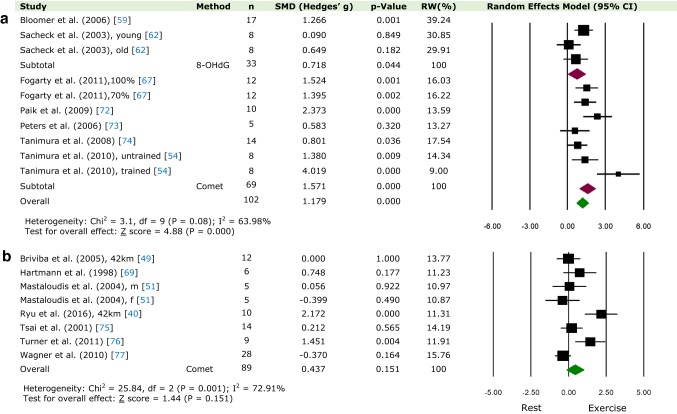
Relative weight (RW) standardised mean difference (SMD) and 95% CI (Hedges’ g adjusted) of DNA damage compared between rest and after an exercise bout at **a** high-intensity exercise (≥ 75% *V*O_2-max_) at time-point 0 (0 h) and 5 (1 day) and **b** long-distance exercise (≥ 42 km) at time-point 0 (0 h) and 1 (15 min–1 h). Values for individual trials and pooled data (random model) are shown and grouped by method of quantification. *m* males, *f* females

DNA damage was increased after high-intensity exercise (≥ 75% *V*O_2-max_), measured at time-point 0 (0 h) and 5 (1 day) (Fig. 3a; SMD = 1.18; 95% CI 0.71, 1.65; *p* < 0.05; heterogeneity: *χ*^2^ = 3.1, *p* = 0.08, *I*^2^ = 63.98%).

#### Long-Distance Exercise (≥ 42 km)

As shown in Fig. 3b, DNA damage was not significantly higher after long- distance (≥ 42 km) exercise at time-point 0 (0 h) and 1 (15 min–1 h) (SMD = 0.48; 95% CI − 0.16, 1.03; *p* = 0.15; heterogeneity: *χ*^2^ = 25.84, *p* = 0.001, *I*^2^ = 72.91%).

### Summary of Findings

**Fig. 4:**
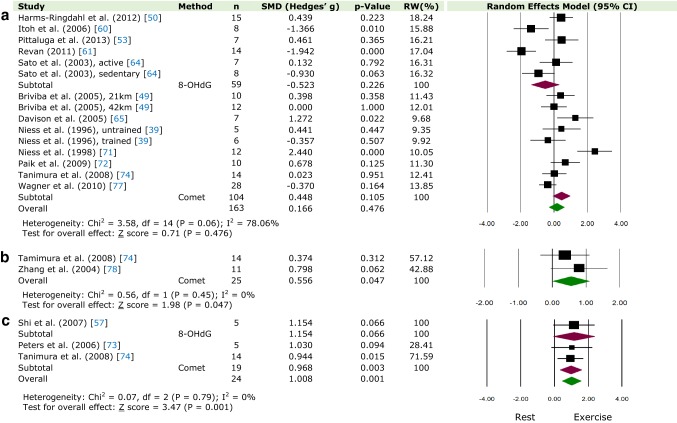
Relative weight (RW) standardised mean difference (SMD) and 95% CI (Hedges’ g adjusted) of DNA damage compared between rest and after an exercise bout at **a** time-point 1 (15 m–1 h), **b** time-point 2 (2 h), and **c** time-point 3 (3 h). Values for individual trials and pooled data (random model) are shown and grouped by method of quantification

Using data from 35 studies and 312 participants, this paper quantitatively demonstrates, for the first time, that DNA damage increases immediately following acute aerobic exercise (Fig. 2). Based on C4ohen’s classification, the effect on DNA damage was large (> 0.8). No significant differences were seen at 1 h (Fig. 4a); however, increased DNA damage was observed from 2 h to 1 day following exercise (Fig. 4b, c; Electronic Supplementary Material Figure S2a, S2b). Similarly, no DNA damage was observed 2 days following exercise (Electronic Supplementary Material Figure S3a) but significantly increased 3 days post-exercise (Electronic Supplementary Material Figure S3b). Furthermore, when comparing the two methods of DNA damage (comet assay and 8-OHdG), a significant difference was observed only in studies using the comet assay, at time-point 0 h, 3 h and 1 day, again with a large effect size. No significant differences were observed 5–28 days post-exercise (Electronic Supplementary Material Figure S4a, S4b and S4c). Finally, when isolating protocols of high intensity (≥ 75% *V*O_2-max_) and long-distance (≥ 42 km), greater DNA damage following exercise was observed only in the former (Fig. 3a, b). However, it should be noted that no long-distance study in our analysis used 8-OHdG as a biomarker for oxidative damage, whereas, in the high-intensity protocols, a mixture of both methods was utilized. As has been suggested [51, 77], DNA damage measured after long-distance exercise (7–10 h race) may not be detected due to the activation of repair mechanisms and increased clearance of damaged cells initiated during the race, which would otherwise not be observed when measured after a shorter exercise protocol. In addition, these processes could be further enhanced due to the intake of antioxidants ingested during the race.

## Discussion

The main purpose of this meta-analysis was to examine the effect of acute exercise on DNA damage. These results suggest that exhaustive exercise leads to increased DNA damage. Acute aerobic exercise, regardless of intensity, seems to produce sufficient stimulus for a greater production of RONS which may evoke damaging effects to DNA. After longer distance events, such as triathlons and ultramarathons, added protection against DNA damage may be offered through the initiation of repair systems and adequate antioxidant intake from food/drinks consumed during such events; however, more studies are needed to confirm this.

### Mechanisms of Free Radical Production During Exercise

Previous work from our laboratory has shown a 63% increase in DNA damage following exercise as well as a greater production of H_2_O_2_ as a function of exercise, compared to rest, indicating a possible mechanism of exercise-induced DNA damage through the increased production of H_2_O_2_ [66]. Moreover, the activation of inflammatory cells such as neutrophils and lymphocytes during exercise, due to muscle tissue damage, can further enhance superoxide production, which can cause direct damage to DNA [75, 80]. In addition, catecholamines released during exercise can be autoxidized and lead to the production of non-radicals such as H_2_O_2_ [81]. Finally, during high-intensity aerobic exercise, tissue ischaemia occurs, resulting in an increased number of hydrogen ions which can in turn react with superoxide anions to produce further RONS [82].

### Free Radical-Induced Damage to DNA/Repair

Although O_2_^·−^ and NO are the primary radical species produced by contracting skeletal muscle, these do not directly cause damage to DNA [14]. Instead, OH^·^ reacts with the different components of DNA, such as DNA bases and the deoxyribose sugar, causing damage either by hydrogen addition or abstraction, producing multiple products, as well as single- and/or double-strand breaks, tandem lesions, and DNA protein cross-links [14, 83]. Among the four DNA bases, guanine has the least reduction potential, and acts as an excellent electron donor and is the most prone to oxidation by OH^·^ [83]. For this reason, the product 8-OHdG is the most popular biomarker of DNA damage in urine and blood samples [12]. Furthermore, compared to guanine, adenine has a greater reduction potential and is not oxidized to the same extent [83]. Just as with guanine, OH^·^ reacts with adenine by adding a hydrogen molecule to its double bonds at specific locations but in a slightly different distribution to that of guanine [83]. The base excision repair pathway is normally activated to repair DNA damage, and this occurs following the activation of a number of enzymes such as DNA glycosylase-1 [84], endonuclease phosphodiesterase, and DNA polymerase [85]. Repair to DNA is almost always controlled by a number of factors such as availability of said enzymes and others such as p53 and RAS [86, 87].

### Hormesis Theory

**Fig. 5:**
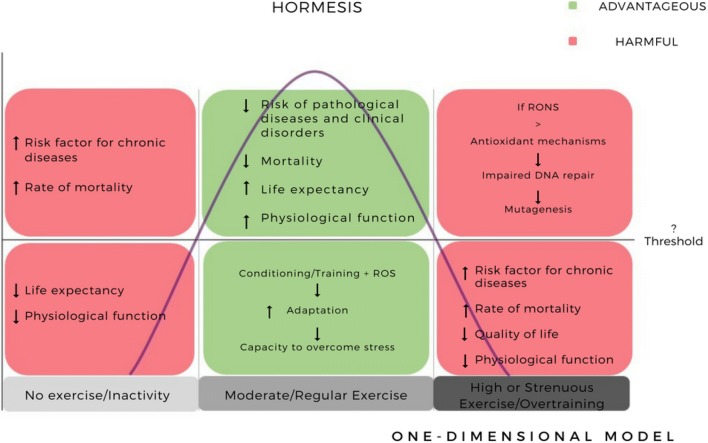
The relationship between exercise and DNA oxidation and its effects explained by the hormesis curve (one-dimensional model). *RONS* reactive oxygen and nitrogen species

The relationship between exercise, RONS, and DNA damage has been explained in the context of the hormesis theory (displayed in Fig. 5) [18]. In toxicology, hormesis refers to an environmental agent’s beneficial effect on a cell or organism at low doses that is otherwise harmful at high doses, creating a bell-shaped curve [88]. In this instance, exercise acts as the stimulus and the subsequent effects of exercise-induced RONS (physiological or pathological) are determined by the dose. Being physically inactive is a major risk factor for numerous chronic diseases and physiological disorders such as cancer, type 2 diabetes, cardiovascular disease, metabolic syndrome, hypertension, and obesity [89–91]. In 2000, physical inactivity in combination with poor diet was the second leading cause of death after tobacco in the US, contributing to 16.6% of total US deaths [92].

Physical inactivity represents one end-point of the hormesis curve, while overtraining and strenuous unaccustomed/unindividualized exercise represents the opposite end-point; both result in a higher risk of disease and decreased physiological function and are mediated by elevated RONS production and oxidative stress [93]. Regular exercise can lead to adaption through up-regulation of molecular and cellular pathways, redox signalling, and antioxidant repair systems, resulting in the enhanced capacity of the organism to overcome greater stress [11, 93]. In addition, exercise training can further extend that adaptive response by ‘stretching’ the capacity to tolerate even higher levels of RONS [93]. Yet, when RONS production outstrips antioxidant defence mechanisms and there is insufficient repair of DNA double-strand breaks, this can cause chromosome instability and gene mutation can occur [15, 94]. However, it is unclear where the threshold limit exists between the beneficial effects of regular exercise and the point of overtraining associated with higher oxidative DNA damage and insufficient repair. This makes the concept of hormesis definitive but narrow. Defining this point is inherently complex due to the heterogeneous variations across individuals based on sex, age, fitness, and exercise intensity and distance. However, along with these, there are even more complex factors (discussed in Sect. 4.5) that influence the degree of the damage and, therefore, the overall effect of the beneficial adaptations and the harmful effects of the two end-points that should be considered.

### One-Dimensional vs Multi-dimensional Model

**Fig. 6:**
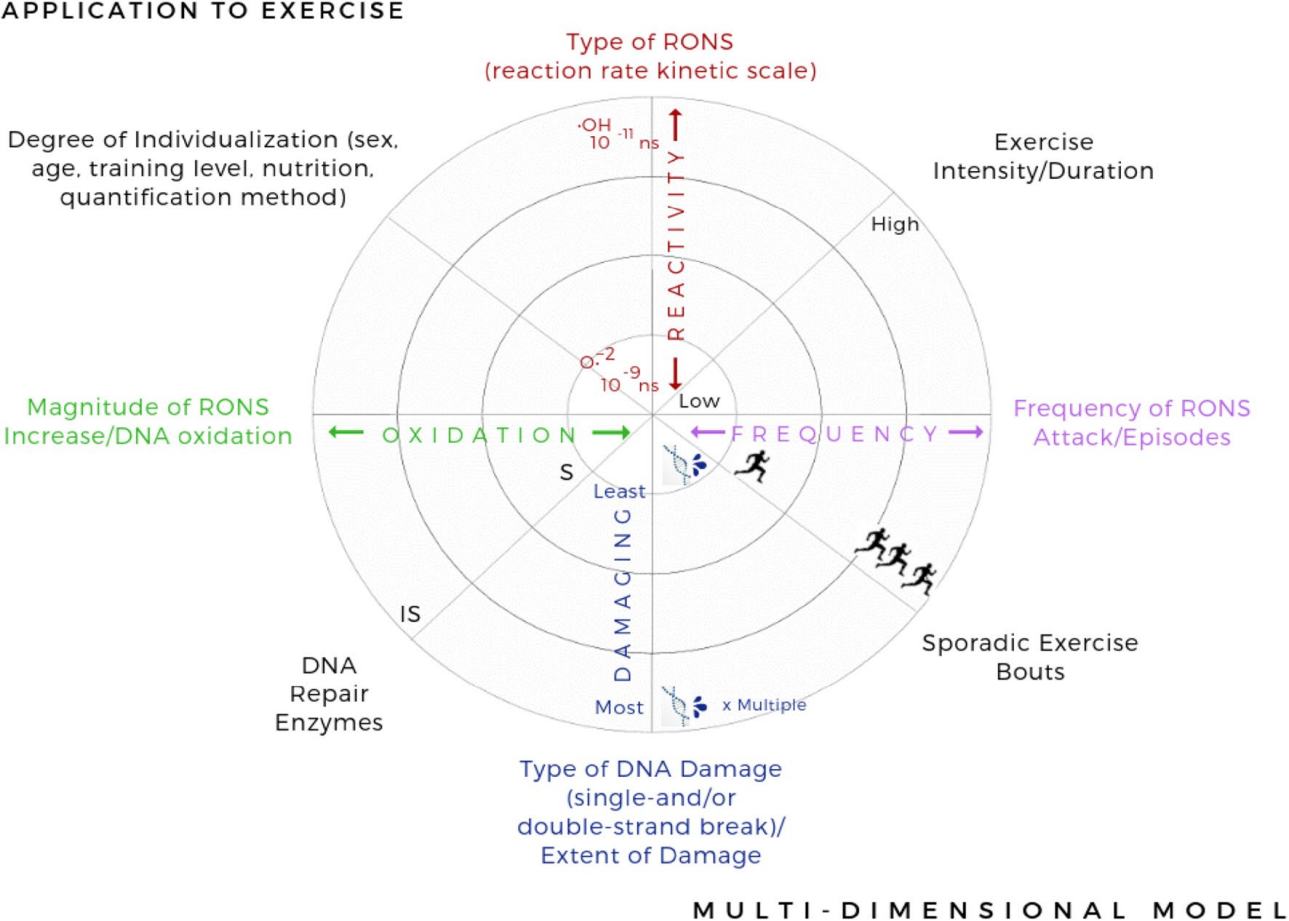
Multi-dimensional model showing multiple factors to be considered when assessing the degree of oxidative damage when applied to the exercise model. *IS* insufficient, *RONS* reactive oxygen and nitrogen species, *S* sufficient

A role for RONS in exercise-mediated adaptations and responses is evident [95]. The concept of hormesis can allow us, to some extent, to understand how the relationship between exercise and DNA oxidation can fit into a bell-shaped curve. However, it only considers levels of RONS/DNA oxidation, rendering it somewhat one-dimensional. While this may be an important factor in explaining the fundamental adaptive responses to exercise, when investigating the extent of DNA oxidative damage, there are multiple factors to consider. We propose three basic factors (instead of only articulating RONS/DNA oxidation) in a more intricate and adaptable multi-dimensional model, visualised in a radar chart starting from the centre (least damaging) to the edge of the circle (most damaging) in a linear scale manner, as shown in Fig. 6. Thus, this proposed multi-dimensional model would consist of the following four factors: (1) type of RONS, ranging from the least reactive (such as O_2_^·−^) to the most reactive radical (such as OH^·^); (2) frequency of RONS attacks/episodes, ranging from one to multiple episodes; (3) type/extent of DNA damage, either single- or double-strand breaks, ranging from the least to the most damaging effect; and (4) magnitude of RONS/DNA oxidation, ranging from lowest to maximum levels of DNA oxidation/RONS increase. When applying this multi-dimensional model to the exercise stimulus, there are four further specific factors to consider: (5) exercise intensity/ distance; (6) exercise frequency; (7) sufficiency of DNA repair enzymes; and (8) degree of individualization (sex, age, training level, and nutrition quantification method) (Fig. 6).

As exercise occurs, adaptive mechanisms are stimulated and these lead to the accentuation of antioxidant enzymes, as a result of training adaptation [96]. However, if multiple individual sporadic bouts of acute, but not regular, exercise occur (effect of overtraining and/or excessive exercise resulting from very high-intensity and/or long-distance exercise), without sufficient rest periods in between, the repair systems most likely fail due to higher oxidative stress resulting from enhanced RONS production [96]. Successful adaptations are thus unlikely and detrimental health outcomes may occur as a consequence. In contrast, individual bouts of exercise with complete recovery in between could revoke any oxidative stress via the antioxidant enzymes which are upregulated within the muscle as a function of training, suggesting that exercise itself can exert an antioxidant effect [97, 98]. In turn, this supports the now established theory that RONS production is in fact a necessary step to stimulate the adaption of the skeletal muscle in response to exercise [19]. Furthermore, the severity of the damage and whether genome stability is being compromised or not depends on the type of damage/oxidation that has occurred to the DNA—base oxidation, single- or double-strand breaks.

Cumulatively, these factors can affect the degree of DNA oxidative damage by causing the least to the most amount of DNA damage and, in turn, possibly creating (individual) different individual thresholds for the end-points of physical inactivity and overtraining and the way the hormesis effect unfolds as a bell-shaped curve. Obviously, a combination of reaching the higher end of the scale in all factors (towards the circumference of the circle) would result in the most harmful kind of oxidative DNA alteration, compared to the lower end of the scale (towards the centre of circle) where the outcome may be less harmful.

This meta-analysis suggests that aerobic exercise leads to an increase in oxidative DNA damage as measured by the comet assay. It is important to elucidate what this means in relation to health outcomes. The literature collectively suggests that a single acute bout of exercise (even of high intensity/long distance) is not likely able to cause any long-term and significantly harmful effects as explained under the hormesis theory. Ironman triathlon studies have shown that well-trained athletes show a large decline in DNA damage post-race. For instance, Mastaloudis et al. reported that DNA damage decreased below baseline levels 2 days after a 50-km ultramarathon [51]. Moreover, an 8% decrease below baseline was observed after 6 days. Similarly, Wagner et al. showed that DNA damage after an Ironman triathlon returned to baseline 5 days after the event, suggesting non-persistent DNA damage [77]. This can be attributed to the up-regulation of repair mechanisms and endogenous antioxidative networks, which indicates that endurance training can enhance the body’s ability to prevent and repair DNA damage, largely by increasing its antioxidant defenses [77]. The non-trained cohorts may not have the added antioxidant protection as a function of adaptive training. Master endurance athletes are also shown to have longer telomere length (TL), a marker of biological age, than non-athlete age-matched controls [99, 100]. Telomeres are responsible for stopping cell division by activating DNA damage recognition systems [101]. TL shortening is attenuated by long-term endurance training, and thus, reduced antioxidant activity and accumulation of RONS may contribute to TL debilitation [100]. Taking this into account, along with the use of different experimental designs, fitness levels, and methods of DNA damage detection at various post exercise TPs, all these factors may affect the degree of the damage and the extent to which it is (efficiently) repaired.

In summary, both the advantageous and harmful effects of exercise-associated adaptations and the two end-points, physical inactivity and overtraining, are caused by non-exposure or repeated exposure to the stimulus (inactivity or repeated exercise bouts) combined with a varying degree of DNA oxidative damage. Whether or not physiological or pathological consequences occur, and to what extent, may depend on all factors mentioned in the multi-dimensional model.

### Strengths and Limitations

This is the first meta-analysis available on DNA damage and exercise. DNA damage was distinguished while performing sensitivity analysis of two of the most common methods of quantification found across studies, the comet assay, and 8-OHdG. The overall risk bias was low, since studies scored well in the quality assessment table. Finally, PRISMA guidelines [33] and Cochrane collaboration recommendations were followed [42].

Some limitations have been identified in the included studies. A number of study data had to be manually extracted from figures due to data not being presented in the text. However, the degree of error should be minimal due to the high accuracy of the software used. Moreover, the sample size for the two quantification methods was not equal, and while this is expected given the variety of study methodologies used is nonetheless noteworthy. This may be the main reason why only studies utilising the comet assay showed significantly greater DNA damage following exercise as opposed to 8-OHdG. However, this could also result due to interlaboratory differences. In 2005, the European Standards Committee on Oxidative DNA Damage found no association between levels of oxidative DNA damage in a sample of 88 healthy males measured by the comet assay and 8-OHdG by HPLC methods in six different laboratories [102]. Therefore, the validity and comparability of different methods of oxidative DNA damage across laboratories may be questioned. Similarly, the number of studies/sample size at all time-points and in the subgroup analysis (high-intensity and long-distance studies) varied, and could explain the difference between observed significance and non-significance between the two protocols.

The authors chose to focus solely on studies that have quantified DNA damage assayed from blood as these represent the most frequently measured in the literature. Nevertheless, we acknowledge that DNA damage can also be determined in urine and muscle. Studies measuring DNA damage following exercise in tissues/specimens other than white blood cells (e.g., muscle and urine) support our data demonstrating that exercise induces DNA damage. Previous work from our laboratory shows an 86% increase, compared to rest, in muscle 8-OHdG concentration following 100 isolated and continuous maximal knee contractions [103]. Moreover, during a 4-day race, urinary 8-OHdG of five super-marathon runners was monitored; where after day 1 (93 km) 8-OHdG increased, on day 2 (120 km), no further increase occurred, while on days 3 and 4 (56 and 59 km, respectively), there was a decrease in 8-OHdG suggesting the likelihood of exercise adaptation and upregulation of antioxidant systems [104]. Similarly, after 8 days of running (30 ± 3 km/day) at a training camp, 8-OHdG measured from urine increased significantly by 26% [105]. Another investigation showed that, after 1 h of cycling at 70% of maximal O_2_ uptake, urinary 8-OHdG was elevated, and this increase remained significant 1 day post-exercise [106].

Furthermore, training status was not distinguished across studies and was only taken into account as to whether it was reported or not in the literature in the quality assessment of this review. There were a few studies using marathons and/or triathlons as the exercise protocol, but most of the investigations did not report the training status of participants. This is important as trained athletes may be less susceptible to oxidative stress due to their enhanced expression of antioxidant enzymes and up-regulation of repair systems, acquired from previous training [77]. Across studies, the time of post-exercise measurement ranged from immediately post-exercise to 28 days following exercise (Table 2). However, in most studies, DNA damage was measured immediately post-exercise. Although this was further investigated by analysis of subsequent time-points, a significant increase at some of those time-points may not have been found, due to a smaller sample size.

### Future Research

A relatively new biomarker has been used recently, the γ-H2AX, to assess DNA double-strand breaks in cancer research [94]. This assay is considered a sensitive method of measuring DNA damage, due to its ability to detect very low levels of double-strand breaks, which the comet assay could not otherwise detect [107]. Lippi et al. reported an increase in DNA injury, associated with running distance and intensity, with γ-H2AX foci analysis in lymphocytes. Amateur runners completed a 5-km, 10-km, 21-km, and 42-km running trial on four separate occasions. The authors observed a small increase in γ-H2AX foci after both 5 km and 10 km of running, a larger increase after 21 km, and an even larger increase after 42 km, indicating a dose-dependent relationship of DNA damage with distance and intensity [108]. This method could represent a salient methodological approach for future research to better address the complexity of exercise and DNA damage. Similarly, although challenging, incorporating direct free radical detection in parallel studies may yield more robust results and sensitive data. Finally, the role of antioxidant supplementation and its potential effects on DNA damage following exercise could be the next focal point of future meta-analyses. As a final practical aspect of performing subsequent meta-analyses, future authors are recommended to include all numeric values in text for easier extraction.

## Conclusions

This systematic review and meta-analysis demonstrates a large increase in DNA damage immediately following an acute exercise bout as well as after 2 h and up to 1 day post-exercise, while such an increase was not evident between 5 and 28 days. Furthermore, only studies using the comet assay showed significance, compared to 8-OHdG. The analysis further showed that high-intensity exercise results in an increase in DNA damage, suggesting that greater DNA damage maybe be positively associated with increasing exercise intensity in a dose-dependent manner, while no significance was observed in the long-distance studies, possibly due to the initiation of repair systems during such events. However, due to limitations discussed and the paucity of evidence for most secondary outcomes, findings should be viewed with a degree of caution. Although an increase in DNA damage occurs after exercise, this is not necessarily a negative outcome per se. Such damage is most likely repaired within 3 days, or likely even sooner, as the long-distance studies may suggest, and thus may be transitory and should not confer any long-term adverse health outcomes on the individual or athlete. However, this will differ across individuals due to variation in individual thresholds, since there are multiple factors to consider as explained (but not limited to) in the multi-dimensional model. The hormesis curve describes, in a somewhat one-dimensional manner, how exercise modulates any advantageous or harmful effects through RONS by increasing DNA oxidation between the two-end points of the curve, physical inactivity and overtraining. Finally, the proposed multi-dimensional model may allow for a better understanding of the complex and multi-factorial relationship between DNA damage and exercise.

## Electronic supplementary material

Below is the link to the electronic supplementary material.

Supplementary material 1 (DOCX 162 kb)

## Data Availability

The data presented in this systematic review are available in the associated studies, and references are provided.
