# Correction to: DNA Damage Following Acute Aerobic Exercise: A Systematic Review and Meta-analysis

**DOI:** 10.1007/s40279-019-01197-4

**Published:** 2019-10-01

**Authors:** Despoina V. Tryfidou, Conor McClean, Michalis G. Nikolaidis, Gareth W. Davison

**Affiliations:** 1 Sport and Exercise Sciences Research Institute, Ulster University, Shore Road, Newtownabbey, Northern Ireland UK; 2 Department of Physical Education and Sports Science at Serres, Aristotle University of Thessaloniki, Serres, Greece

## Correction to: Sports Medicine 10.1007/s40279-019-01181-y

Page 17, Fig. 2: The following figure, which previously read:


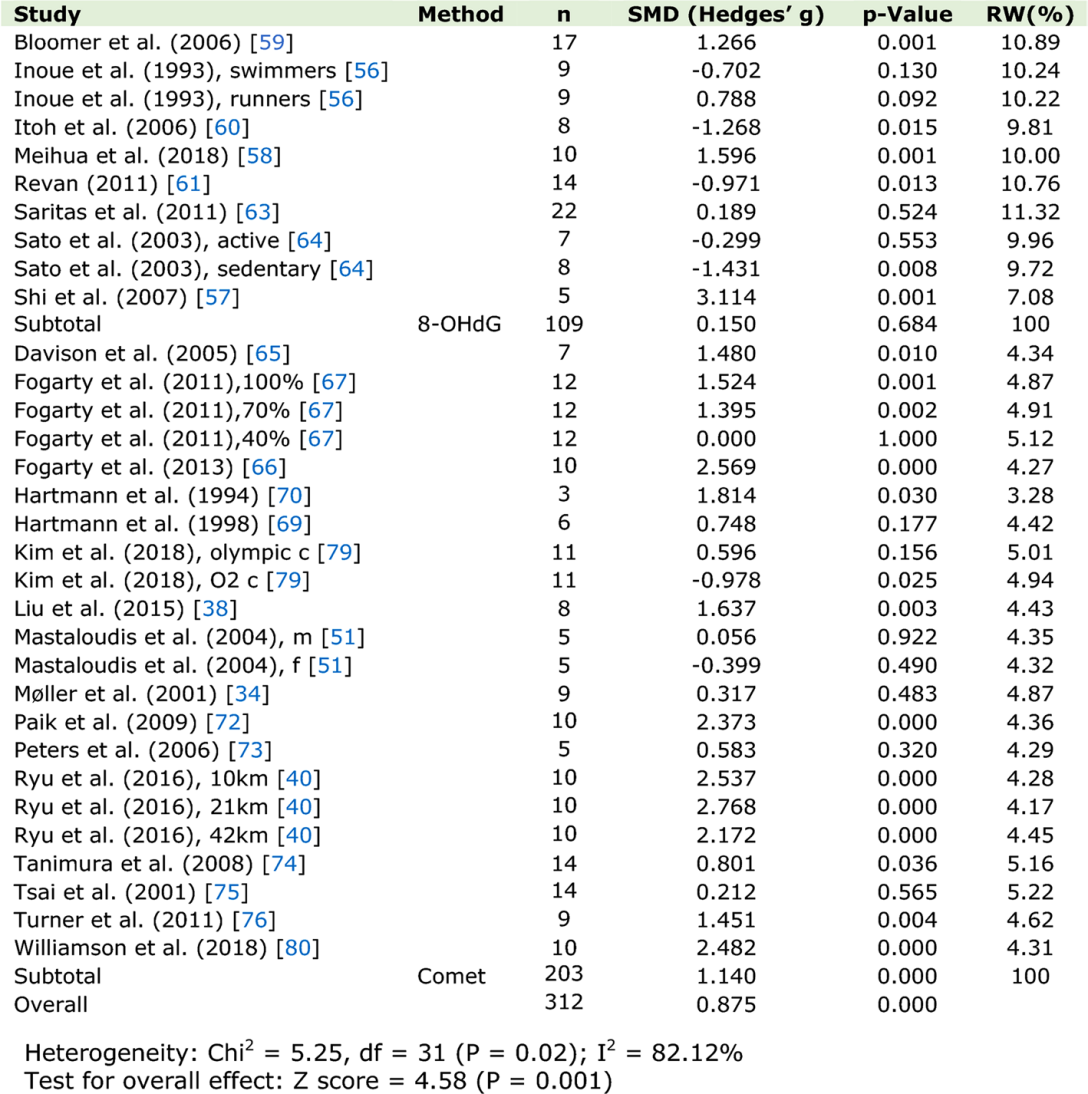


should read:


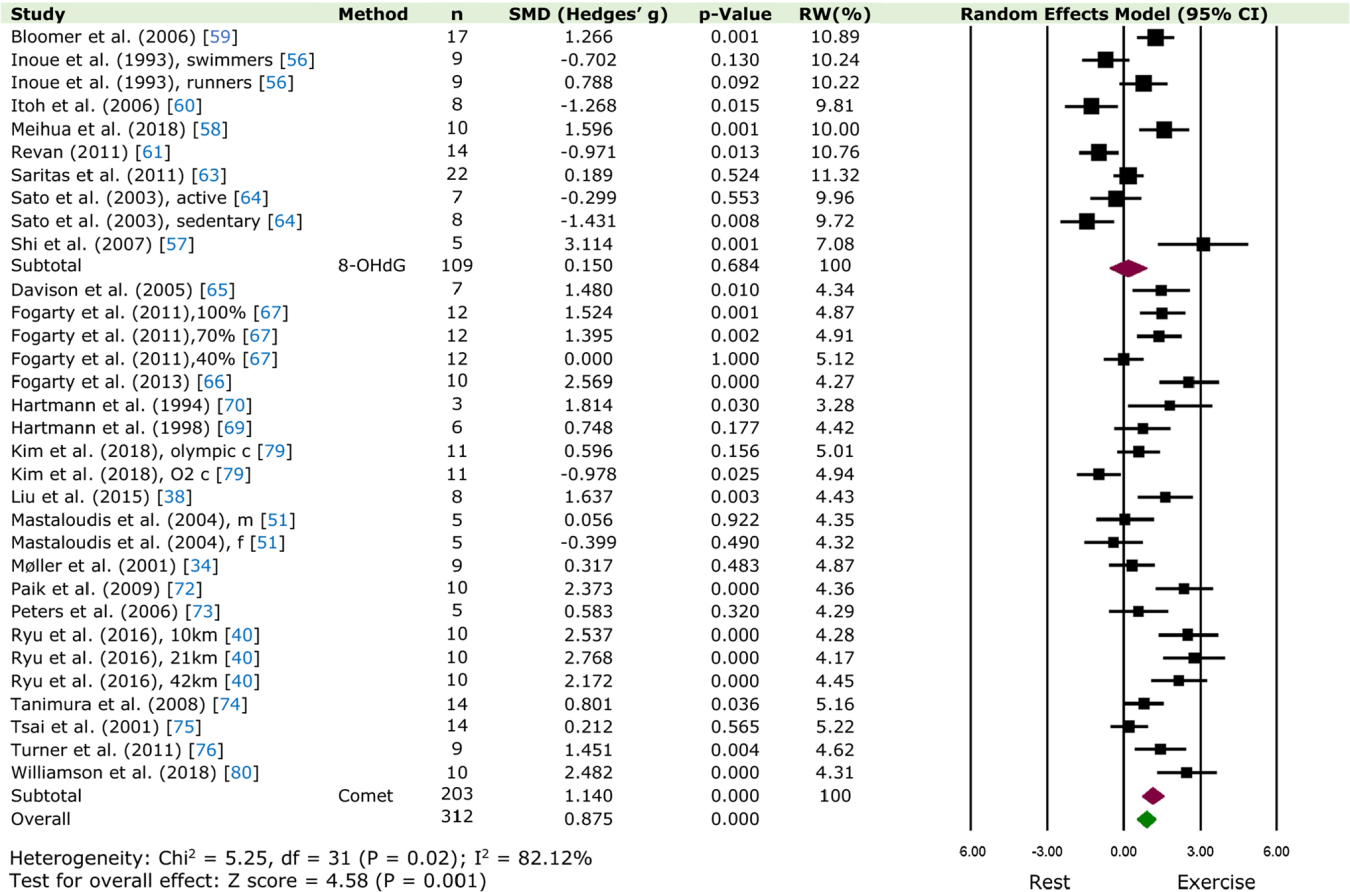


Page 18, Fig. 3: The following figure, which previously read:


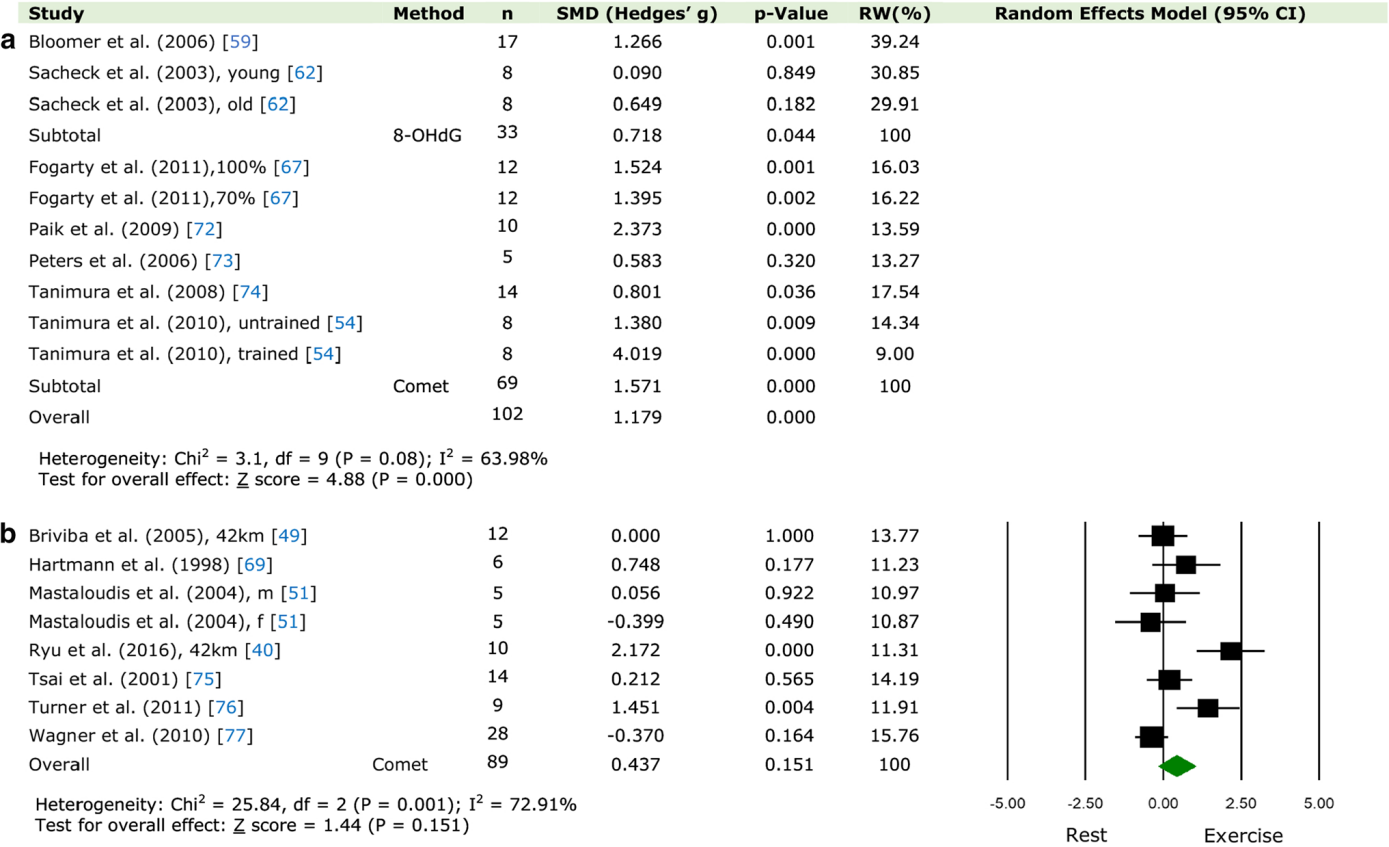


should read:


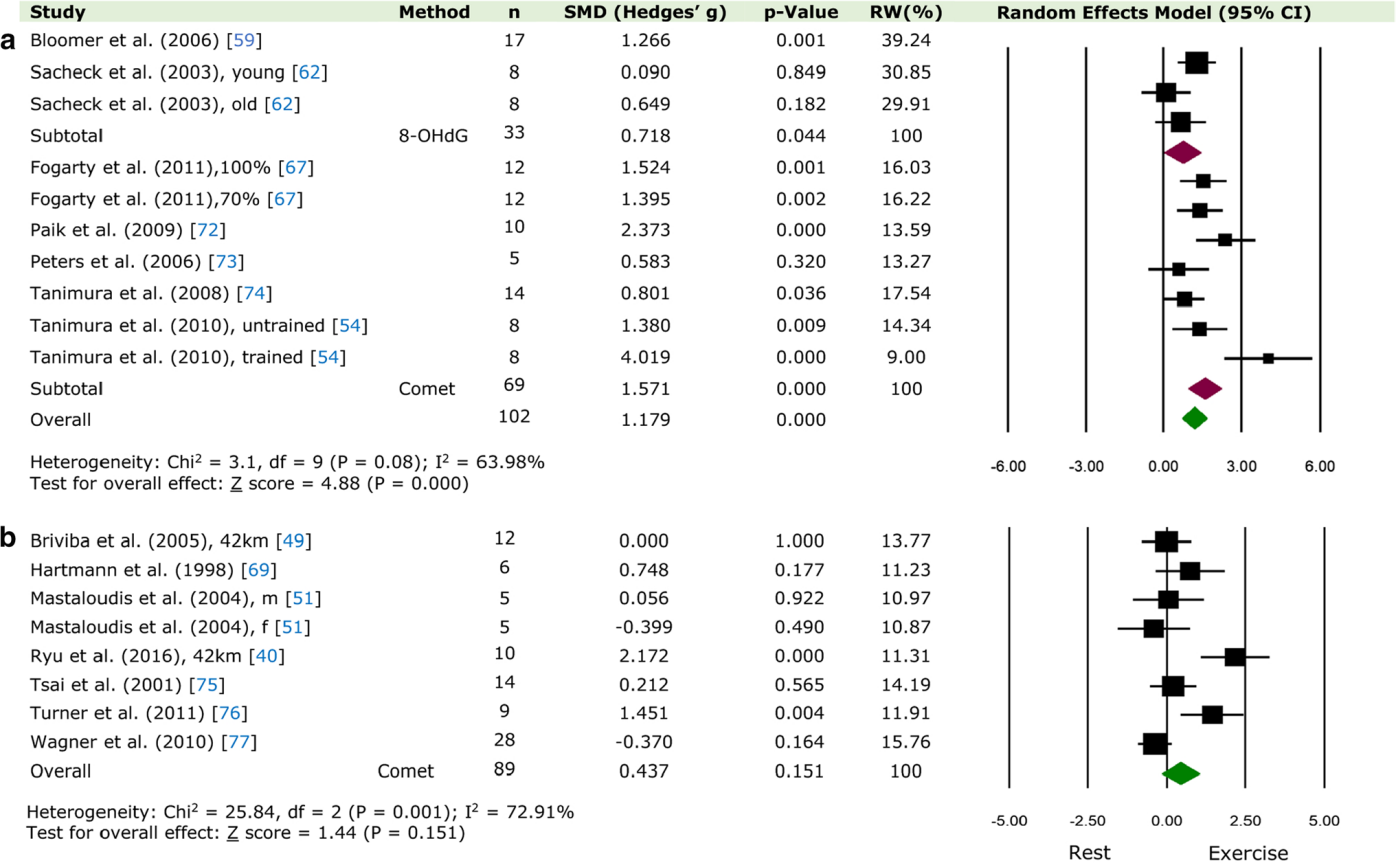


The original article has been corrected.

