# Supplementary material for: DNA Damage Following Acute Aerobic Exercise: A Systematic Review and Meta-analysis
Source: Sports Med. 2019 Sep 16;50(1):103–27. doi: 10.1007/s40279-019-01181-y (PMC6942015; doi:10.1007/s40279-019-01181-y)
Supplement: Supplementary file 1 — Supplementary material 1 (DOCX 162 kb) [file 40279_2019_1181_MOESM1_ESM.docx]

**
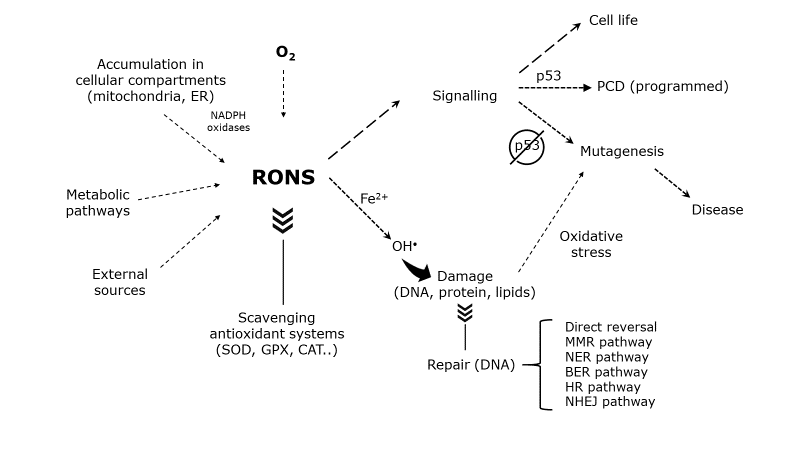
**

**Electronic Supplementary Figure S1.** RONS production, scavenging and DNA damage repair pathways. Adapted [28].

Abbreviations: ER, endoplasmic reticulum; RONS, reactive oxygen and nitrogen species; NADPH, nicotinamide adenine dinucleotide phosphate; SOD, superoxide dismutase; GPX, glutathione peroxidase; CAT, catalase; O_2,_ dioxygen; OH^•^, hydroxyl radical; PCD, physiological cell death; MMR, mismatch repair; NER, nucleotide excision repair; BER, base excision repair; HR, homologous recombination; NHEJ, non-homologous end joining.

**Electronic Supplementary Table S1.** Studies excluded with reasons.

| **Study** | **Reasons for exclusion** |
| --- | --- |
| Díaz-Castro et al. (2012) | The study used urine sampling to report DNA damage. |
| Mota et al. (2010) | No exercise protocol was implemented; only measures of aerobic fitness through *V*O_2-max_ test. |
| Mergener et al. (2009) | No exercise protocol was implemented; only physical activity levels reported. |
| Atli et al. (2013) | Exercise protocol was a 3-day football tournament; neither specification of duration nor intensity. |
| Shockett et al. (2016) | Study used cell-free mitochondrial DNA. |
| Cash et al. (2014) | No exercise protocol was implemented; only physical activity levels reported. |
| Leonardo-Mendonça et al. (2014) | Exercise protocol included multiple training days. |
| Kim et al. (2010) | Exercise protocol was a 9-week training program. |
| Tomasello et al. (2012) | No exercise protocol was implemented. |
| Fogarty et al. (2013) | Exercise was 100 isolated knee extension contractions. |
| Sarmiento et al. (2016) | Exercise protocol consisted of 10 resistance type body-building strenuous exercises. |
| Cuevas et al. (2005) | Exercise was anaerobic. |
| Mrakic-Sposta et al. (2015) | Capillary blood was used. |
| Bloomer et al. (2005) | Exercise protocol consisted of both aerobic and anaerobic exercise. |
| Gray et al. (2014) | Exercise was 200 repetitions of eccentric knee contractions. |

| **Study** | **Reasons for exclusion** |
| --- | --- |
| Shockett et al. (2016) | Plasma cell-free mitochondrial DNA was used. |
| Atamaniuk et al. (2004) | Plasma cell-free DNA concentrations were used. |
| Arazi et al. (2015) | Exercise protocol consisted of traditional vs. cluster resistance exercise loading patterns. |
| Ra et al. (2013) | Exercise was eccentric elbow flexor exercises. |
| Palazzetti et al. (2003) | Exercise involved 4 weeks of overload training. |
| Giacomo et al. (2009) | No exercise protocol was implemented. |
| Demirbağ et al. (2005) | Participants were referred for evaluation of suspected coronary artery disease. |
| Neubauer et al. (2010) | Reported the same population as in another study included in the review. |

**Electronic Supplementary Table S1.** Studies excluded with reasons.

| **Study** | **Method** | **n** | **SMD (Hedges’ g)** | **p-Value** | **RW(%)** |
| --- | --- | --- | --- | --- | --- |
| Hartmann et al. (1994) [70] |  | 3 | 2.817 | 0.007 | 19.23 |
| Zhang et al. (2004) [78] |  | 11 | 1.772 | 0.000 | 37.97 |
| Tanimura et al. (2008) [74] |  | 14 | 0.621 | 0.099 | 42.80 |
| Overall | Comet | 28 | 1.480 | 0.010 | 100 |


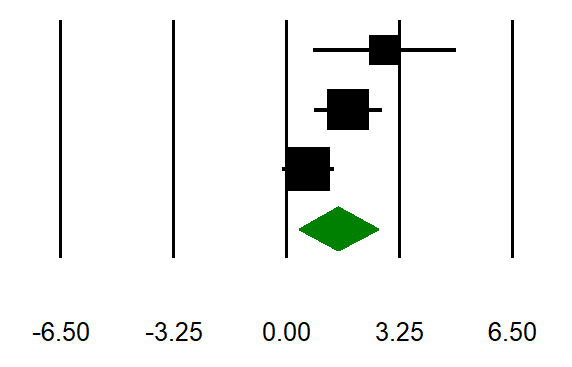


**a**

**Random Effects Model (95% CI)**

Heterogeneity: Chi^2^ = 6.13, df = 2 (P = 0.047); I^2^ = 67.37%

Test for overall effect: Z score = 2.58 (P = 0.01)

| Pittaluga et al. (2013) [53] |  | 7 | 1.279 | 0.021 | 12.16 |
| --- | --- | --- | --- | --- | --- |
| Sacheck et al. (2003), young [62] |  | 8 | 0.090 | 0.849 | 13.03 |
| Sacheck et al. (2003), old [62] |  | 8 | 0.649 | 0.182 | 12.89 |
| Itoh et al. (2006) [60] |  | 8 | -1.290 | 0.014 | 12.49 |
| Saritas et al. (2011) [63] |  | 22 | 0.347 | 0.245 | 14.69 |
| Sato et al. (2003), active [64] |  | 7 | 0.707 | 0.172 | 12.56 |
| Sato et al. (2003), sedentary [64] |  | 8 | -2.372 | 0.000 | 11.35 |
| Shi et al. (2007) [57] |  | 5 | 1.673 | 0.014 | 10.82 |
| Subtotal | 8-OHdG | 73 | 0.141 | 0.720 | 100 |
| Hartmann et al. (1994) [70] |  | 3 | 5.250 | 0.001 | 3.94 |
| Hartmann et al. (1995) [68] |  | 8 | 3.383 | 0.000 | 6.46 |
| Hartmann et al. (1998) [69] |  | 6 | 2.583 | 0.001 | 6.50 |
| Mastaloudis et al. (2004), m [51] |  | 5 | -0.261 | 0.650 | 6.99 |
| Mastaloudis et al. (2004), f [51] |  | 5 | -0.101 | 0.860 | 7.00 |
| Møller et al. (2001) [34] |  | 9 | -0.305 | 0.499 | 7.29 |
| Niess et al. (1996), u [39] |  | 5 | 4.285 | 0.000 | 5.39 |
| Niess et al. (1996), t [39] |  | 6 | 1.586 | 0.011 | 6.86 |
| Sardas et al. (2012), rowers [55] |  | 12 | 11.335 | 0.000 | 3.86 |
| Sardas et al. (2012), PE s [55] |  | 11 | 13.253 | 0.000 | 3.12 |
| Tanimura et al. (2010), u [54] |  | 8 | 1.380 | 0.009 | 7.10 |
| Tanimura et al. (2010), t [54] |  | 8 | 4.019 | 0.000 | 6.19 |
| Tsai et al. (2001) [75] |  | 14 | 1.127 | 0.004 | 7.40 |
| Turner et al. (2011) [76] |  | 9 | 1.329 | 0.008 | 7.17 |
| Wagner et al. (2010) [77] |  | 28 | 0.606 | 0.025 | 7.62 |
| Zhang et al. (2004) [78] |  | 11 | 2.232 | 0.000 | 7.10 |
| Subtotal | Comet | 148 | 2.468 | 0.000 | 100 |
| Overall |  | 221 | 1.113 | 0.000 |  |

**b**


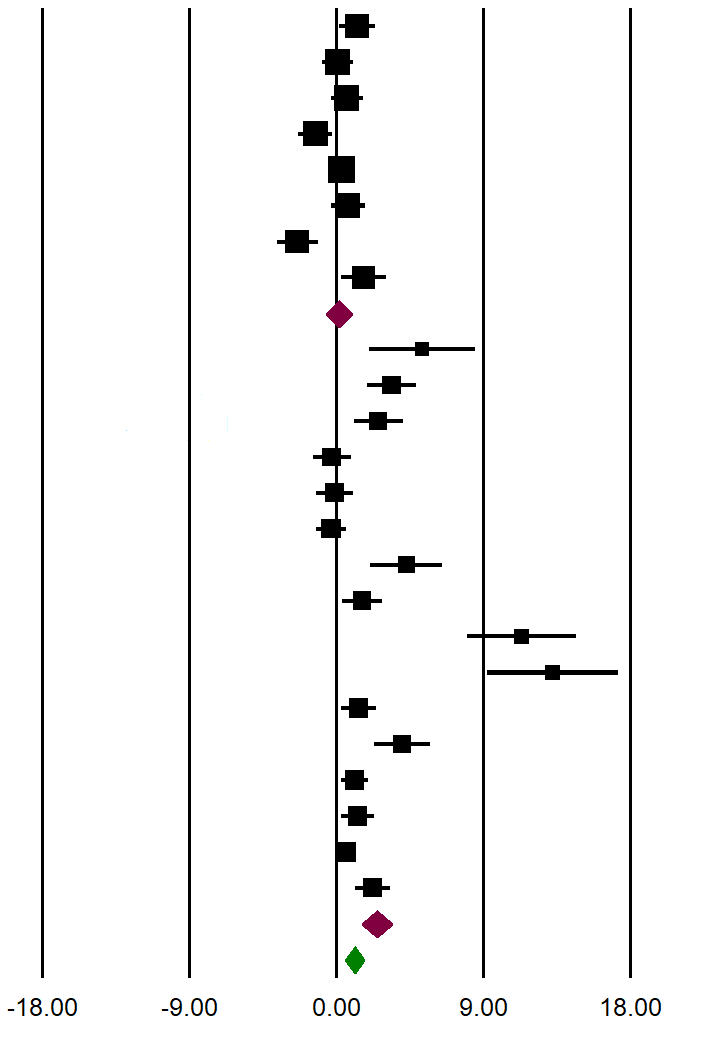


**Electronic Supplementary Figure S2.** Relative weight (RW) standardised mean difference (SMD) and 95% CI (Hedges’ g adjusted) of DNA damage compared between rest and after an exercise bout at **(a)** time-point 4 (4-6h) and **(b)** time-point 5 (1d). Values for individual trials and pooled data (Random Model) are shown and grouped by method of quantification. Abbreviations: m, males; f, females; u, untrained; t, trained; PE s, physical education students.

Exercise

Rest

Heterogeneity: Chi^2^ = 14.58, df = 23 (P = 0.000); I^2^ = 88.34%

Test for overall effect: Z score = 3.71 (P = 0.000)

| **Study** | **Method** | **n** | **SMD (Hedges’ g)** | **p-Value** | **RW(%)** |
| --- | --- | --- | --- | --- | --- |
| Sato et al. (2003), active [64] |  | 7 | 0.585 | 0.254 | 50.96 |
| Sato et al. (2003), sedentary [64] |  | 8 | -3.071 | 0.000 | 49.04 |
| Subtotal | 8OHdG | 15 | -1.208 | 0.509 | 100 |
| Hartmann et al. (1994) [70] |  | 3 | 1.555 | 0.050 | 16.58 |
| Hartmann et al. (1998) [69] |  | 6 | 1.848 | 0.005 | 19.18 |
| Mastaloudis et al. (2004), m [51] |  | 5 | -0.738 | 0.214 | 20.33 |
| Mastaloudis et al. (2004), f [51] |  | 5 | -0.190 | 0.741 | 20.76 |
| Møller et al. (2001) [34] |  | 9 | -0.369 | 0.416 | 23.15 |
| Subtotal | Comet | 28 | 0.338 | 0.497 | 100 |
| Overall |  | 43 | 0.231 | 0.630 |  |

| Hartmann et al. (1994) [70] |  | 3 | -0.284 | 0.666 | 8.39 |
| --- | --- | --- | --- | --- | --- |
| Hartmann et al. (1998) [69] |  | 6 | 7.625 | 0.000 | 2.36 |
| Kim et al. (2018), olympic c [79] |  | 11 | 0.221 | 0.591 | 11.92 |
| Kim et al. (2018), O2 c [79] |  | 11 | 0.485 | 0.245 | 11.84 |
| Mastaloudis et al. (2004), m [51] |  | 5 | 0.112 | 0.845 | 9.53 |
| Mastaloudis et al. (2004), f [51] |  | 5 | 0.531 | 0.363 | 9.37 |
| Ryu et al. (2016), 10km [40] |  | 10 | 0.684 | 0.122 | 11.45 |
| Ryu et al. (2016), 21km [40] |  | 10 | 0.406 | 0.349 | 11.59 |
| Ryu et al. (2016), 42km [40] |  | 10 | 0.307 | 0.476 | 11.62 |
| Tsai et al. (2001) [75] |  | 14 | 1.384 | 0.001 | 11.93 |
| Overall | Comet | 85 | 0.627 | 0.022 | 100 |

| Hartmann et al. (1994) [70] |  | 3 | -0.071 | 0.913 | 25.59 |
| --- | --- | --- | --- | --- | --- |
| Hartmann et al. (1998) [69] |  | 6 | 3.970 | 0.000 | 21.11 |
| Mastaloudis et al. (2004), m [51] |  | 5 | -0.065 | 0.909 | 26.70 |
| Mastaloudis et al. (2004), f [51] |  | 5 | -0.419 | 0.469 | 26.60 |
| Overall | Comet | 19 | 0.691 | 0.380 | 100 |


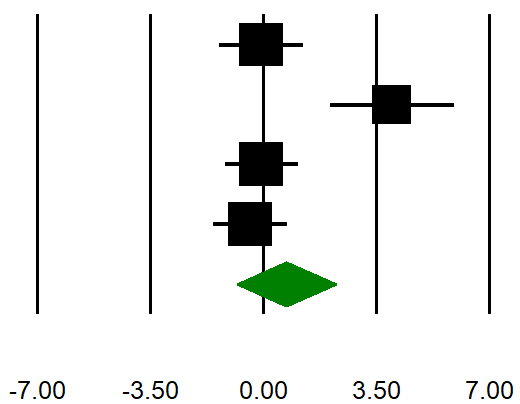

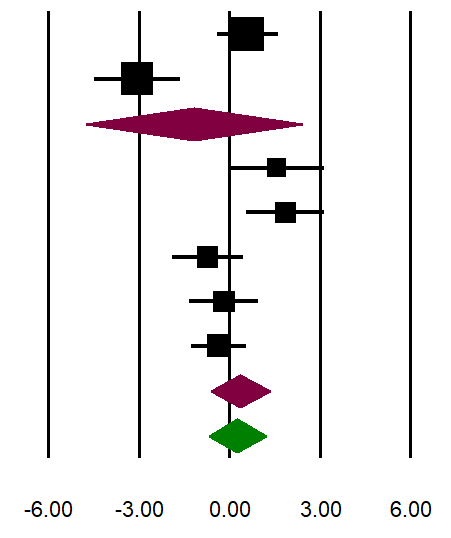

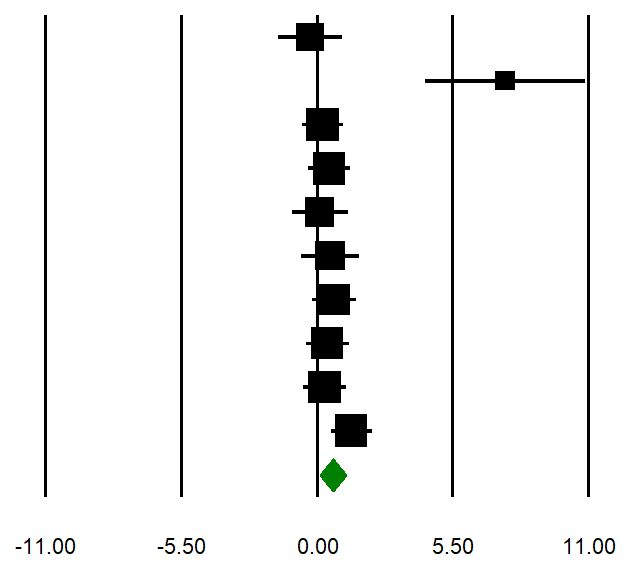


**Electronic Supplementary Figure S3.** Relative weight (RW) standardised mean difference (SMD) and 95% CI (Hedges’ g adjusted) of DNA damage compared between rest and after an exercise bout at **(a)** time-point 6 (2d), **(b)** time-point 7 (3d) and **(c)** time-point 8 (4d). Values for individual trials and pooled data (Random Model) are shown and grouped by method of quantification. Abbreviations: c, course; m, males; f, females.

Heterogeneity: Chi^2^ = 16.58, df = 3 (P = 0.001); I^2^ = 81.91%

Test for overall effect: Z score = 0.88 (P = 0.38)

Heterogeneity: Chi^2^ = 25.98, df = 9 (P = 0.002); I^2^ = 65.36%

Test for overall effect: Z score = 2.29 (P = 0.022)

Heterogeneity: Chi^2^ = 0.66, df = 6 (P = 0.41); I^2^ = 82.17%

Test for overall effect: Z score = 0.48 (P = 0.63)

Rest

Exercise

**b**

**c**

**a**

**Random Effects Model (95% CI)**

| **Study** | **Method** | **n** | **SMD (Hedges’ g)** | **p-Value** | **RW(%)** |
| --- | --- | --- | --- | --- | --- |
| Hartmann et al. (1998) [69] |  | 6 | 2.468 | 0.001 | 20.44 |
| Mastaloudis et al. (2004), m [51] |  | 5 | -0.025 | 0.965 | 24.27 |
| Mastaloudis et al. (2004), f [51] |  | 5 | -0.616 | 0.294 | 23.87 |
| Wagner et al. (2010) [77] |  | 28 | -0.117 | 0.657 | 31.42 |
| Overall | Comet | 44 | 0.315 | 0.548 | 100 |


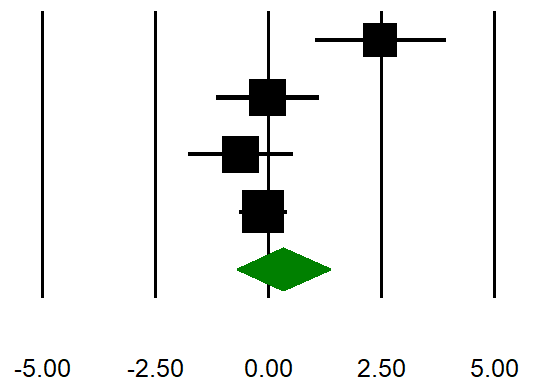


**a**

**Random Effects Model (95% CI)**

Heterogeneity: Chi^2^ = 12.58, df = 3 (P = 0.006); I^2^ = 76.15%

Test for overall effect: Z score = 0.6 (P = 0.548)

| Mastaloudis et al. (2004), m [51] |  | 5 | -0.116 | 0.839 | 23.49 |
| --- | --- | --- | --- | --- | --- |
| Mastaloudis et al. (2004), f [51] |  | 5 | -0.108 | 0.850 | 23.50 |
| Tsai et al. (2001) [75] |  | 14 | 1.745 | 0.000 | 26.68 |
| Turner et al. (2011) [76] |  | 9 | -0.205 | 0.649 | 26.33 |
| Overall | Comet | 33 | 0.359 | 0.491 | 100 |


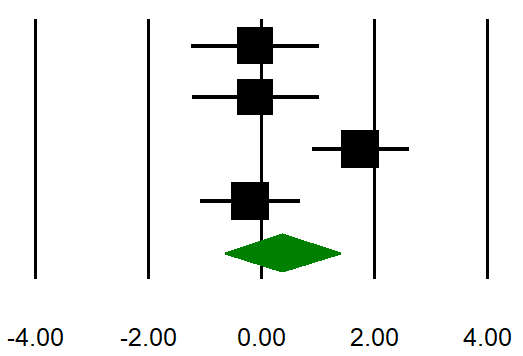


**b**

Heterogeneity: Chi^2^ = 12.92, df = 3 (P = 0.005); I^2^ = 76.78%

Test for overall effect: Z score = 0.69 (P = 0.491)

| Tsai et al. (2001) [75] |  | 14 | 0.679 | 0.073 | 32.78 |
| --- | --- | --- | --- | --- | --- |
| Turner et al. (2011) [76] |  | 9 | 0.314 | 0.487 | 29.34 |
| Wagner et al. (2010) [77] |  | 28 | -0.555 | 0.039 | 37.88 |
| Overall | Comet | 51 | 0.105 | 0.801 | 100 |


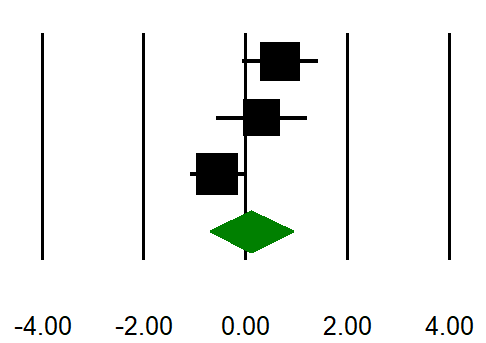


**c**

Heterogeneity: Chi^2^ = 7.9, df = 2 (P = 0.02); I^2^ = 74.67%

Test for overall effect: Z score = 0.25 (P = 0.801)

Exercise

Rest

**Electronic Supplementary Figure S4.** Relative weight (RW) standardised mean difference (SMD) and 95% CI (Hedges’ g adjusted) of DNA damage compared between rest and after an exercise bout at **(a)** time-point 9 (5d), **(b)** time-point 10 (6d-7d) and **(c)** time-point 11 (14d-28d). Values for individual trials and pooled data (Random Model) are shown and grouped by method of quantification. Abbreviations: m, males; f, females.
